# Analysis of ovarian cancer immune cell profile identifies immunosuppressive states associated with adverse clinical attributes and survival times

**DOI:** 10.1371/journal.pone.0346746

**Published:** 2026-04-20

**Authors:** Ana Moscoso, Navid Mohammad Mirzaei, Leili Shahriyari

**Affiliations:** 1 Department of Mathematics and Statistics, University of Massachusetts Amherst, Amherst, Massachusetts, United States of America; 2 Department of Epidemiology, Mailman School of Public Health, Columbia University, New York, New York, United States of America; Xiangya Hospital Central South University, CHINA

## Abstract

The ovarian tumor microenvironment (TME) is highly immunosuppressive, limiting immunotherapy effectiveness. We investigated whether the composition, ratios, and polarization of infiltrating immune cells provide prognostic value in ovarian cancer (OC). Our analysis revealed that immune ratios, including CD8/Treg and CD8/CD4, were more predictive of survival than absolute CD8 + , CD4 + , or Treg levels. Prior studies have reported associations between higher CD8/Treg ratios and improved responses to immune checkpoint inhibitors, highlighting the importance of effector–regulator balance; however, immunotherapy response was not evaluated in this cohort. Additionally, macrophage polarization proved to be crucial: pro-tumor M2-macrophages were associated with vascular invasion, persistent tumor, and worse survival, while higher naïve M0-macrophage levels predicted improved outcomes. Surprisingly, anti-tumor M1-macrophages lacked prognostic significance, suggesting possible benefits in preserving an M0-macrophage pool. Neutrophil infiltration, though relatively uncommon, correlated with poor survival, supporting reports that neutrophils suppress T-cell responses. Immune infiltration was linked to aggressive features such as vascular invasion, reflecting both heightened recognition and compensatory recruitment of suppressive populations. Unsupervised clustering identified four immune-defined subtypes, with worse survival in clusters enriched for M2-macrophages and CD4 + T-cells and depleted in M0-macrophages, particularly in advanced disease. Overall, our findings highlight the prognostic value of immune ratios, macrophage polarization, and neutrophil activity in OC, suggesting new avenues for risk stratification and future therapeutic investigation.

## Introduction

Ovarian cancer is responsible for 3.4% of all diagnosed cancer cases and 4.7% of cancer deaths in females worldwide [1]. In 2022, there were about 324,603 new cases of ovarian cancer globally, while in the same year, there were about 206,956 ovarian cancer deaths [2]. Most ovarian cancer patients are diagnosed in advanced stages due to ambiguous early symptoms and limited screening efficacy [3,4]. The ovarian tumor microenvironment (TME) is typically “immunologically cold” [5,6]. This refers to a tumor that does not provoke a strong immune response, largely due to a lack of infiltrating immune cells like T cells and the abundant presence of immunosuppressive factors. These include cytokines such as IL-6, TNF-α, and IL-8; immune checkpoint inhibitors of CD8 + T cells; and the presence of pro-tumor macrophages and regulatory T cells [7]. This makes treatment approaches, especially immunotherapy, more challenging for this cancer type.

Immunotherapy is a type of cancer treatment that utilizes a diverse set of strategies to deploy or modify the patient’s immune system to control or eradicate a disease such as cancer. It has revolutionized the treatment of several cancers and is currently under investigation to find more efficient approaches for ovarian cancer, including immune checkpoint inhibitors, dendritic cell vaccines, and adoptive cell transfer [8–11]. However, clinical responses have been modest, mostly hindered by immune evasion mechanisms and the heterogeneity of immune infiltration [6,12,13]. Furthermore, given the variability of immune frequency among patients, there has been a need for patient-specific approaches.

Extensive biological and translational studies have focused on the ovarian tumor microenvironment and its immune composition [14,15]. The tumor microenvironment hosts a complex network of interactions between cytokines and malignant, stromal, endothelial, and immune cells that shape tumor progression and therapeutic response [16]. Several studies have shown that high infiltration of cytotoxic CD8 + T cells is associated with improved survival, whereas enrichment of immunosuppressive cells, such as regulatory T cells (Tregs) and M2-polarized macrophages, correlates with poor outcomes [15,17,18]. Neutrophils and myeloid-derived suppressor cells (MDSCs) further contribute to immune evasion by promoting angiogenesis and dampening T-cell activity [19,20]. In addition, cytokines like IL-6 and VEGF secreted by tumor and stromal cells sustain chronic inflammation, vascular remodeling, and immune suppression [21,22]. These findings underscore that ovarian cancer prognosis is strongly shaped by immune composition, and they confirm the importance of dissecting immune interactions within the tumor microenvironment to identify therapeutic strategies and biomarkers for patient stratification.

Computational tools have proved helpful in revealing valuable information about the tumor microenvironment that is challenging or impossible to obtain via the available empirical methods. Bulk deconvolution algorithms, such as CIBERSORTx [23], have made it possible to infer the composition of immune cells within the tumor microenvironment. Single-cell RNA-sequencing studies have provided higher-resolution information by mapping the heterogeneity of cell types and creating an atlas of the tumor microenvironment [24]. Additionally, there has been a recent increase in spatially resolved transcriptomic approaches focusing on cell-cell interactions and signaling niches within the tumor microenvironment [25,26]. Statistical methods can be applied to the outputs of these approaches to identify potential biomarkers and to highlight pathways that are critical for patient survival or tumor progression. Mathematical modeling also provides a more downstream strategy: by integrating such data, models can predict tumor dynamics and evaluate the outcomes of alternative therapeutic or biological hypotheses [27–29]. Despite progress, most models of tumor–immune interactions simplify the ovarian tumor microenvironment by focusing on single immune cell types and often neglect patient-level heterogeneity. This limits their ability to capture the full complexity of immune infiltration patterns observed in clinical cohorts and reduces their translational predictive value.

Evidence indicates that immune cell balance might be prognostic in ovarian cancer [15,18,30]. Additionally, systematic approaches to stratify patients by their immune infiltration profiles remain scarce. Here, we present a detailed investigation of the immune composition within the tumor microenvironment of 295 ovarian cancer samples from the Cancer Genome Atlas (TCGA) cohort. We use CIBERSORTx to estimate immune cell proportions from bulk RNA-seq data and apply statistical analyses to examine correlations between immune infiltration, clinical features, and patient survival. We also compare our CIBERSORTx bulk RNA-seq deconvolution results with immune cell estimates derived from single-cell RNA-seq data [31] to validate the accuracy and robustness of our inference. While unsupervised clustering of immune features has provided valuable insights in other cancers [32], its application to ovarian cancer remains limited. In this study, we extend these approaches by employing K-means clustering and subsequent survival analysis to identify immune-based patient subgroups and assess their prognostic relevance. Together, our results shed light on the primary clinical and immunological factors responsible for ovarian cancer immunosuppression and demonstrate a significant dependence on these factors when stratifying patients by their immune profiles and assessing their survival outcomes.

## Materials and methods

### Data

The gene expression data sets are obtained from The Cancer Genome Atlas (TCGA) Ovarian Cancer (OV) [33]. Patients’ normalized RNA-seq data were downloaded from the UCSC Xena web portal [34], and their corresponding clinical data were downloaded from the GDC data portal [35]. Additional clinical data from the TGCA study were downloaded from cBioportal [36]. These data were accessed on December 12th, 2024 and authors do not have access to information that could identify individual participants during or after data collection. We acquired a cohort of 295 total patients from these databases. Key characteristics of this cohort are given in Table 1.

**Table 1:** Clinical characteristics of TCGA cohort of ovarian cancer patients.

Characteristic	Category	Number of patients
**Age at diagnosis**	< 50	65
	≥ 50	230
**Vital status**	Alive	119
	Dead	176
**Neoplasmic histological classification**	G2	42
	G3	243
	GX	5
	GB	1
	Not reported	4
**Stage**	IIB	5
	IIC	19
	IIIA	7
	IIIB	13
	IIIC	217
	IV	31
	Not reported	3
**Tissue invasion**	Lymphovascular	83
	No lymphovascular	41
	Vascular	57
	No vascular	41
	Not reported	73
**Therapy**	Pharmaceutical	278
	Radiation	17
**Tumor status**	Tumor free	92
	With tumor	203

### Statistical analysis

We use the $\chi^{2}$ test to analyze the relationship between categorical variables in this study. We also employ the Mann–Whitney–Wilcoxon test from the SciPy library [37] to detect any significant difference between groups of continuous variables, such as immune fractions, age, and immune score. For comparisons involving more than two groups, such as differences in immune cell fractions across immune-defined clusters, we use the Kruskal–Wallis rank-sum test implemented in the SciPy library [38]. To account for multiple comparisons across immune cell types, p-values from Kruskal–Wallis tests are adjusted using the Benjamini–Hochberg [39] false discovery rate (BH-FDR) correction, and adjusted p-values less than 0.05 are considered statistically significant. Similar to Trang et al. [27], we utilize Pearson correlation and the corresponding p-value to study the correlation between different levels of immune infiltration.

### Clustering and immune profiles

To calculate the patients’ immune cell fractions, we use a linear digital cytometry method called CIBERSORTx [23] on the gene expression data. This software offers improved accuracy compared to other similar packages [40]. We then apply an unsupervised K-means clustering algorithm to cluster tumors based on the percentage of infiltrating immune cells. The K-means algorithm separates the samples into groups of equal variance by minimizing the distance between samples in the clusters and the center of the clusters [41]. The elbow method was then used to find the best value for K (i.e., the number of clusters) [41]. We employ the K-means package from Python’s sklearn.cluster to carry out these steps [42].

We also performed a Principal Component Analysis (PCA) to visualize the variance structure of immune cell proportions and assess how well the K-means algorithm differentiates ovarian tumor samples according to their immune composition. PCA reduces high-dimensional data into a small number of orthogonal components that capture the majority of variance across samples [43]. The analysis was implemented using the PCA module from Python’s sklearn.decomposition package [42].

### Immune score

For this study, we incorporate the immune scores, which represent the infiltration of immune cells in tumor tissue. This measurement estimates the prognosis of cancer patients based on the immune cells that infiltrate the tumor and its surrounding areas. A higher immune score represents a high level of infiltrating immune cells in the tumor [44]. We adopt the ESTIMATE algorithm, which calculates the immune scores of all samples in the cohort [44]. ESTIMATE uses single-sample gene set enrichment analysis (ssGSEA) [45] to calculate the immune score from gene expression data.

### Survival analysis

We employ Kaplan-Meier survival analysis to calculate the overall survival rate based on the levels of immune cells per cluster. The Kaplan-Meier survival curve is defined as the probability of surviving for a given length of time [46]. A P-value of < 0.05 was considered statistically significant by the log-rank test [47]. We used the packages from lifelines logrank test and KaplanMeierFitter [48].

## Results

We analyze the immune landscape and clinical attributes of ovarian cancer patients in the TCGA cohort. The following section presents the key findings from this analysis, highlighting both significant associations and emerging patterns.

We began by examining the relationship between vital status at the time of follow up and clinical characteristics, as well as immune cell fractions in primary tumors at the time of diagnosis. When looking at age at diagnosis vs. vital status, the dead patients were older overall (p-value = 8.602e-03) (Fig 1A). We also tested all immune cells against vital status; the two that showed statistically significant differences were M2 macrophages and Tregs (Fig 1B and 1C). Other attributes do not exhibit any significance. We have shared them in the supplementary materials. See Supplement Figures 1-19 in S1 File.

**Fig 1:**
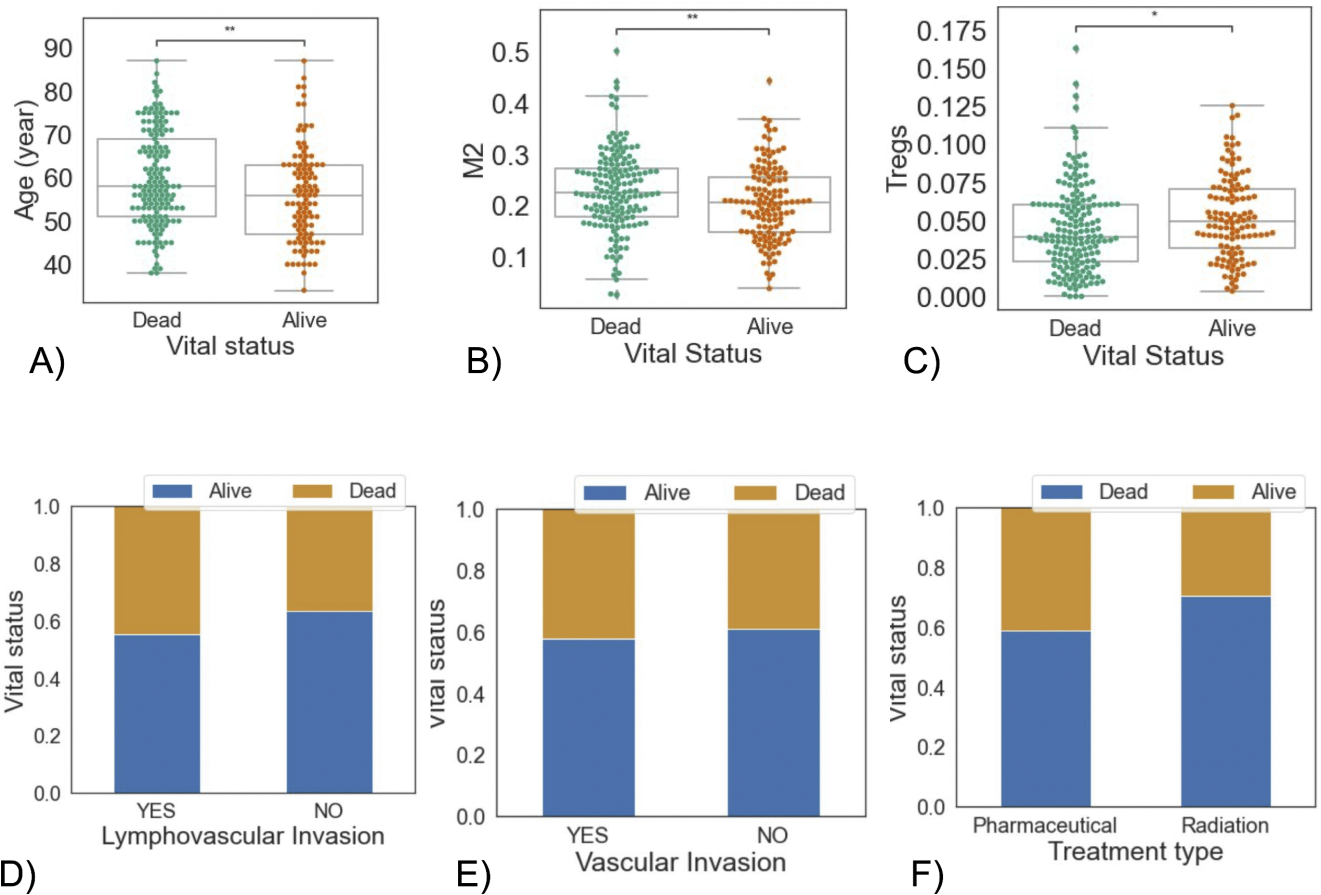
Associations between vital status and immune or clinical features. Boxplots of **(A)** patients’ age at diagnosis, **(B)** M2 cell frequency, and **(C)** T-regulating cells vs vital status. Boxplots indicate the median (center line) and interquartile range (IQR; box), with whiskers extending to 1.5 × IQR. Individual points represent individual patients. Asterisks indicate significant differences from the Mann-Whitney-Wilcoxon test. (ns: no significance, *:0.01<p≤0.05,**:0.001<p≤0.01). Bar plots of patients’ vital status portion vs **(D)** lymphovascular invasion, **(E)** vascular invasion, and **(F)** treatment type. Bars represent relative frequencies within each group.

We also observed more deaths among patients who exhibit lymphovascular invasion, vascular invasion, and those who received pharmaceutical therapy instead of radiation (Fig 1D, 1E, and 1F).

**Fig 2:**
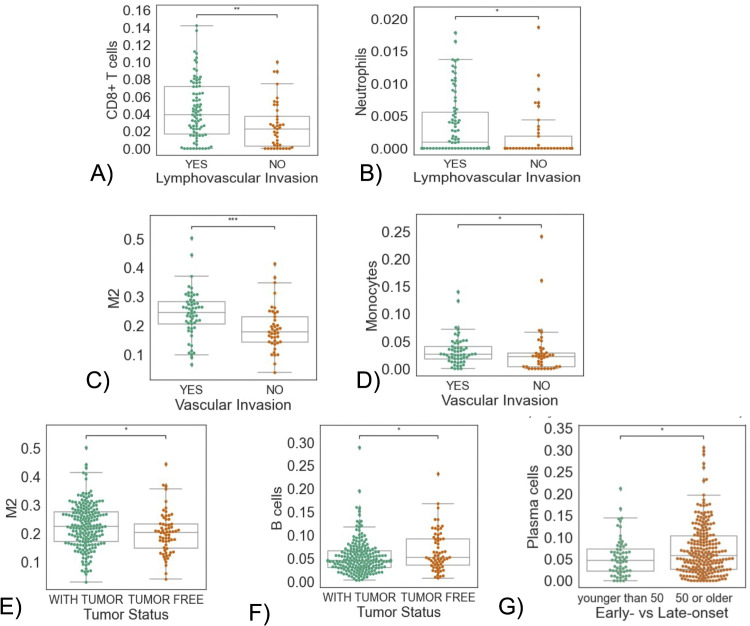
Associations between immune cell infiltrates and clinical features. **(A)** T cells CD8 vs. lymphovascular invasion, **(B)** Neutrophils vs. lymphovascular invasion, **(C)** M2 macrophages against vascular invasion, **(D)** Monocytes vs. vascular invasion, **(E)** M2 Macrophages against follow-up tumor status, **(F)** B cells against tumor status, and **(G)** Plasma cells vs. onset age. Boxplots indicate the median (center line) and interquartile range (IQR; box), with whiskers extending to 1.5 × IQR. Asterisks indicate significant differences from the Mann-Whitney-Wilcoxon test. (ns: no significance, *:0.01<p≤0.05,**:0.001<p≤0.01,***:0.0001<p≤0.001.

All immune cells were examined in relation to age at diagnosis, treatment type, vascular invasion, lymphovascular invasion, and follow-up tumor status. Here, we only show the attributes with significant differences for each immune cell type (Fig 2). For other non-significant attributes refer to Supplemental Figures 1-19 in S1 File. Tumors with lymphovascular invasion show significantly higher CD8 + T-cell and neutrophil infiltration (Fig 2A and 2B). On the other hand, vascular invasion is accompanied by markedly higher levels of M2 macrophages and Monocytes (Fig 2C and 2D). Greater M2 infiltration is significantly associated with worse tumor status at follow-up, unlike B cells, which show the opposite effect (Fig 2E and 2F). Monocyte infiltration is more noticeable in early-onset (younger than 50) compared to late-onset cancers (older than 50) (see Fig 2G).

We then carried out a survival analysis for all patients based on their immune cells, using median, mean, upper, and lower quantile values. For other non-significant attributes refer to Supplemental Figures 20-23 in S1 File.

**Fig 3:**
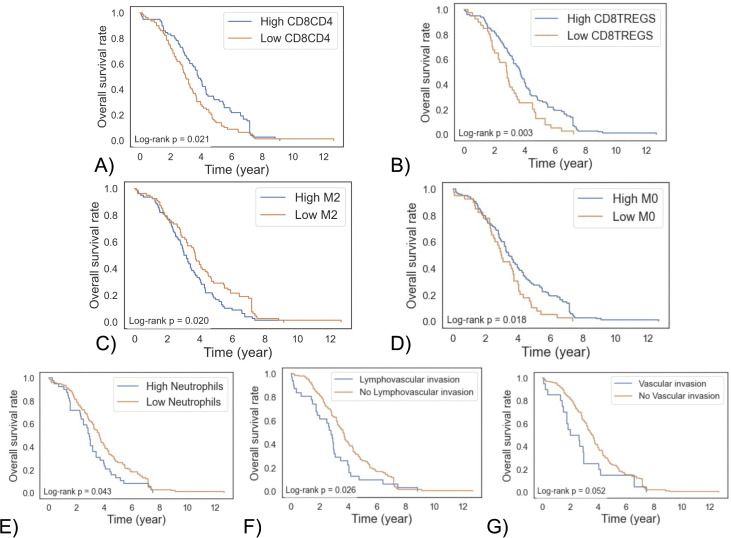
Kaplan–Meier survival analysis based on immune cell ratios, infiltration levels, and invasion status in ovarian cancer patients. **(A)** Overall survival based on high vs. low CD8/CD4 ratio, using median values as cut-off, **(B)** overall survival between based on high vs. low CD8/Tregs ratio, using lower quantile values as cut-off, **(C)** overall survival curves between based on high vs. low M2 Macrophages, using median values as cut-off, **(D)** overall survival based on high vs. low M0 Macrophages using lower quantile values as cut-off, **(E)** overall survival based on high vs. low neutrophil infiltration, using higher quantile values as cut-off, **(F)** overall survival based on the presence of lymphovascular invasion, and **(G)** overall survival based on the presence of vascular invasion.

Significant differences in patient survival were observed based on specific immune cell ratios and infiltration levels (Fig 3). Patients with high versus low CD8/Treg and CD8/CD4 ratios (using median values as cutoffs) showed distinct survival outcomes (p = 0.04 and p = 0.02, respectively; Fig 3A–3B). Similarly, a significant survival difference was found between patients with high and low levels of M2 macrophages, based on median stratification (p = 0.02; Fig 3C), as well as for M0 macrophages when using the 25th percentile as a threshold (p = 0.02; Fig 3D). Elevated neutrophil levels, stratified by upper quantile cutoffs, were also associated with reduced survival (Fig 3E). In contrast, no significant survival differences were observed for other immune cells, age at diagnosis, immune scores, or M1/M2 macrophage ratios. While cross-sectional vital status proportions at the last follow-up appear similar between groups (1 D–E), Kaplan–Meier analysis reveals differences in the timing of mortality, highlighting worse overall survival among patients with lymphovascular or vascular invasion (Fig 3F and 3G).

Based on these findings, we hypothesized that the composition of the immune microenvironment—particularly the balance between effector and suppressive cell types—plays a decisive role in shaping ovarian cancer outcomes. In line with the immunosuppressive phenotype observed in many patients, we identified features such as elevated M2 macrophages and neutrophils that were linked to poor prognosis. In contrast, higher levels of naïve (M0) macrophages appeared protective. Moreover, the association of adverse conditions such as lymphovascular and vascular invasion to immune infiltration was heavily leaning towards a more suppressive environment. To further probe these patterns, we classified patients in the TCGA cohort according to their immune infiltration profiles, with the goal of identifying which immune-related signatures, ratios, and cell states are most strongly associated with favorable versus unfavorable prognosis.

**Fig 4:**
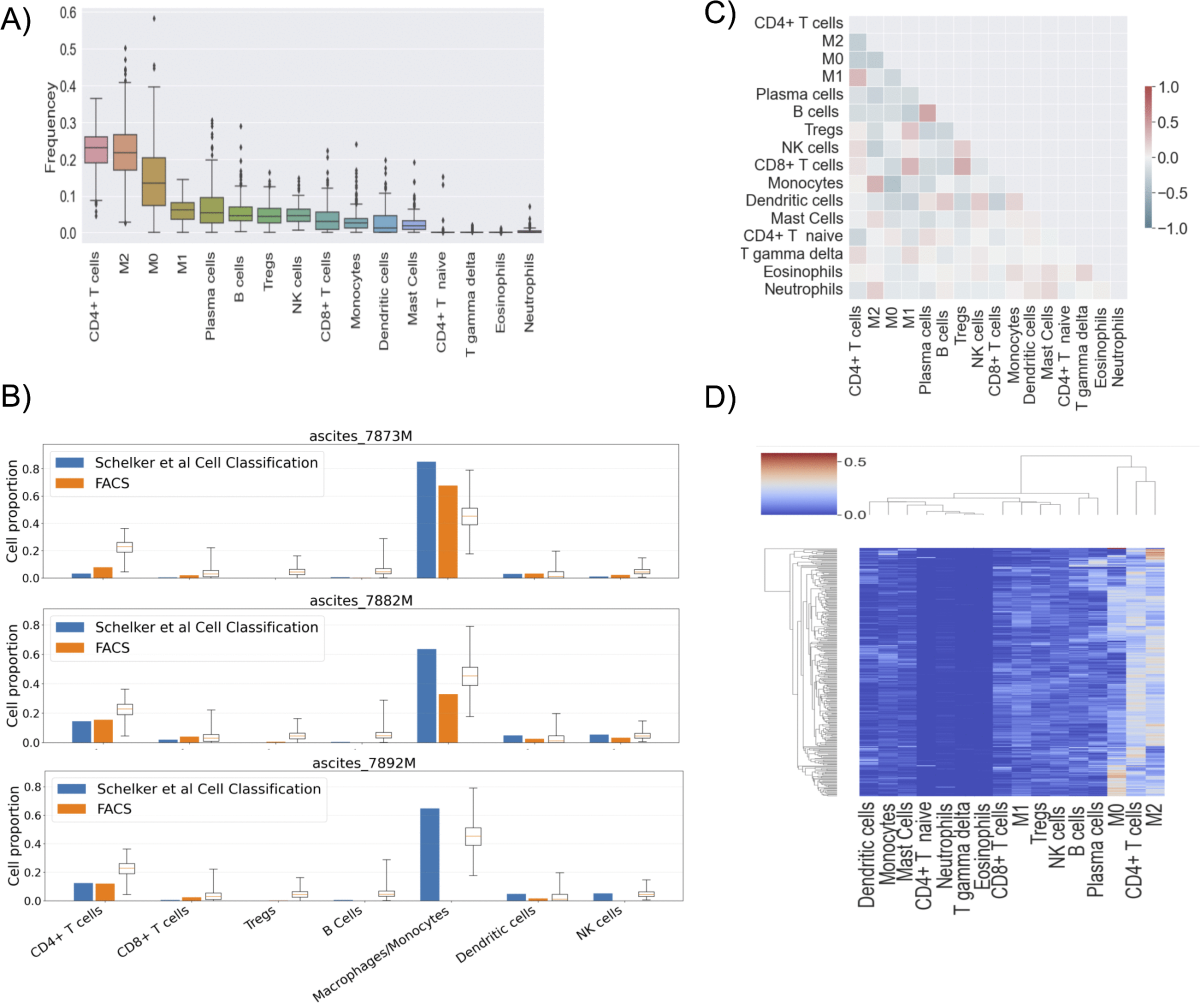
Overview of immune cell distribution and correlations. **(A)** The boxplot of immune cells’ fractions. **(B)** Comparison between cell proportions of three ovarian ascites patient samples evaluated by cell type classification (blue bars), Fluorescence-activated Cell Sorting (FACS) (orange bars) both from Schelker et al [31] and our bulk RNA-seq deconvolution results using CIBERSORTx (box plots). Note in sample 7892M Schelker et al do not provide a FACS measurement for Macrophages/Monocytes. **(C)** Correlations between the immune cell frequencies. **(D)** Hierarchical clustering of estimated immune cell infiltration.

We first started by investigating the overall abundance of each immune cell. Fig 4A shows the immune cell portions resulting from the gene expression deconvolution by CIBERSORTx. Supplemental Figure 24 A in S1 File shows stacked bar plot of immune frequencies per patients. These bulk RNA-seq deconvolution results were generated in this study using CIBERSORTx applied to primary ovarian tumor transcriptomic data, whereas the FACS-derived immune cell proportions shown in Fig 4B (orange bars) were obtained from previously published experimental datasets from Schelker et al [31] and are presented for comparison and validation. M2 macrophages are one of the most abundant immune cells, and we have already established their importance in tumor and vital status and vascular invasion. More interestingly, the other cell types, such as B cells, Tregs, CD8 + , Monocytes, Plasma cells, and Neutrophils, are not as dominant, although their overall prognostic value was acknowledged earlier. Our cell proportion hierarchy is similar to the cellular composition reported by Schelker et al. [31] (see Fig 4B). Notably, the immune cell proportions reported by Schelker et al. were derived from single-cell RNA sequencing of ovarian cancer ascitic fluid samples, whereas our analysis was performed on primary tumor tissue. Although ascitic fluid and primary tumor tissue represent distinct tumor microenvironments, the overall immune cell composition hierarchy remained consistent across both datasets. All of Schelker’s results fall within our box plot ranges and our inferred ranking (Macrophages ≫ T cells > B/NK/Dendritic cells) is quantitatively consistent with these single-cell findings, further supporting the biological plausibility of our CIBERSORTx deconvolution results. Together, these comparisons demonstrate that our computational deconvolution results are consistent with independently generated experimental measurements, supporting the validity of our inferred immune cell composition.

The overall correlations between immune cell fractions (Fig 4C) reveal several notable patterns. Positive associations are observed between Tregs and CD8 + T cells (r = 0.38), M1 macrophages and CD8 + T cells (r = 0.33), M2 macrophages and monocytes (r = 0.34), and B cells and plasma cells (r = 0.42). In contrast, negative correlations are found between monocytes and M0 macrophages (r = −0.39), CD4 + T cells and M2 macrophages (r = −0.32), and between M0 and M2 macrophages (r = −0.31). Moreover, T regs are somewhat negatively correlated with B cells (r = −0.23). Given the intricate correlations observed among immune cell types, particularly those identified as essential in our previous experiment, we performed unsupervised clustering analysis. The results revealed a primary cluster comprising CD4 T cells and M2 macrophages, which subsequently associates with M0 macrophages (Fig 4D).

**Fig 5:**
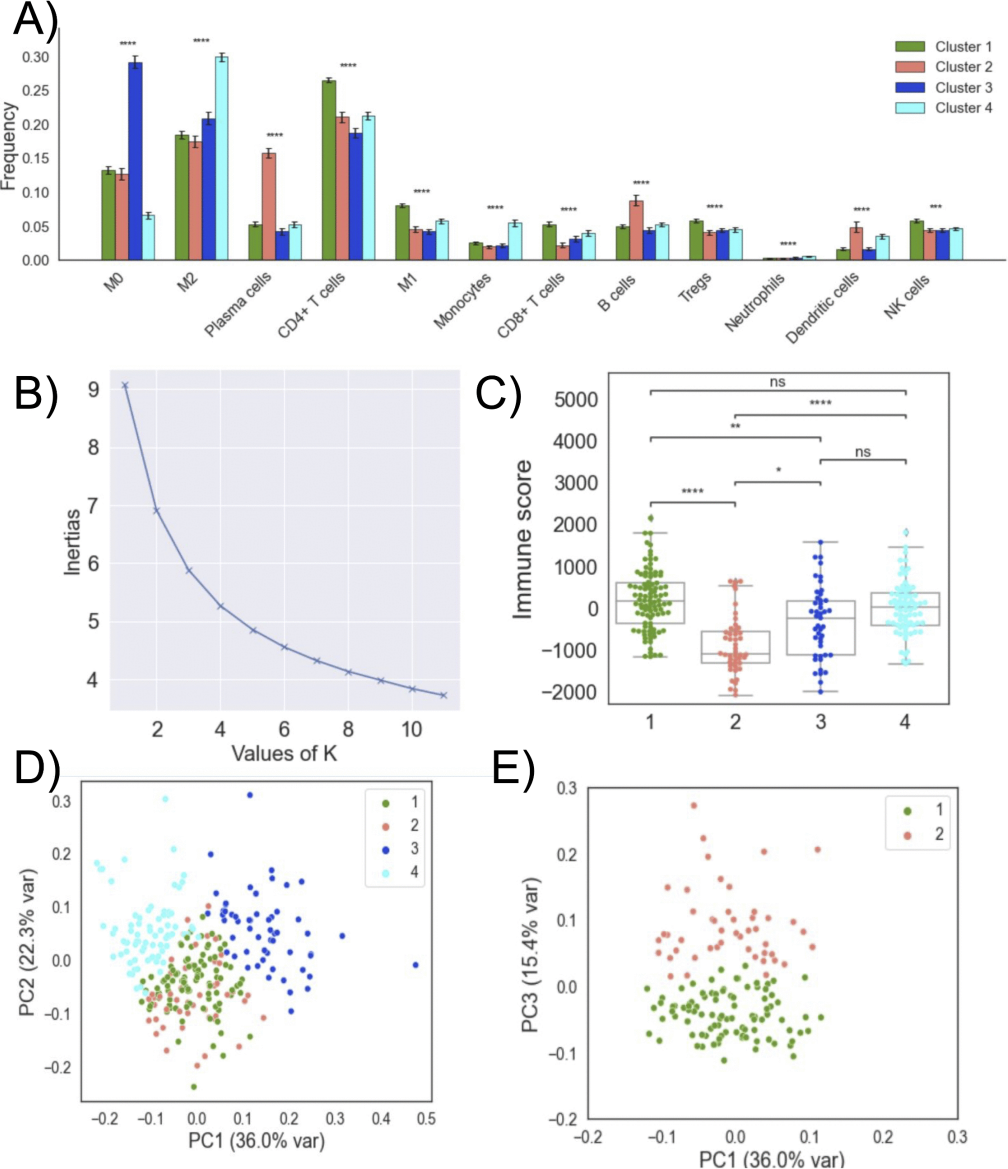
Overview of immune cell clustering. **(A)** For each immune cell type, distributions were compared across clusters 1–4. Asterisks above each cell type indicate the FDR-adjusted global Kruskal–Wallis significance level (ns: no significance, *:0.01<p≤0.05,**:0.001<p≤0.01,***:0.0001<p≤0.001. **(B)** Elbow plot for k-means clustering. **(C)** The box plots of patients’ ESTIMATE immune score in each cluster. **(D)** PC1–PC2 projection of all clusters. **(E)** PC1–PC3 projection restricted to clusters 1 and 2.

K-means clustering analysis identified four different immune infiltration profiles among all OC patients (Supplemental Figure 24 B in S1 File). The choice of k = 4 was supported by the elbow method (Fig 5B), where the reduction in within cluster sum of squares began to plateau beyond four clusters, indicating that additional clusters provided only marginal improvements in model fit. Fig 5A show the statistically significant differences in immune frequencies among clusters. The most prominent differences across clusters are observed in the relative abundance of M0 and M2 macrophages (Fig 5A). Cluster 1 shows the most CD4 cells among all clusters, while Cluster 2 has the highest number of plasma cells. Clusters 1 and 2 display relatively moderate levels of M0 and M2 macrophages. Cluster 3, however, possesses the highest portion of M0 macrophages, whereas Cluster 4 is the most enriched in M2 macrophages. These results suggest predominantly macrophage-driven cluster profiles.

Principal component analysis of immune cell proportions further supports the ability of the K-means clustering algorithm to differentiate ovarian tumors based on distinct immune compositions (Fig 5D–5E). The PC1–PC2 plot (D) shows clear separation among clusters with partial overlap between clusters 1 and 2, whereas the PC1–PC3 projection (E) reveals these two clusters as fully separated, indicating finer immune distinctions captured along PC3. Supplemental Figures 24 C-D in S1 File show PC1-PC3 across all clusters and PC1-PC2-PC3 across all clusters.

We further investigate the clustering by assessing their differences in immune scores (Fig 5C) Our analysis shows that Cluster 2 has the lowest immune score of all clusters (Fig 5C). Mann-Whitney-Wilcox test shows a significant difference between clusters 2 and all other clusters, as well as between cluster 1 and cluster 3. There was no significant difference among clusters in age at diagnosis and days to death (Supplemental Figures 24 E-F in S1 File).

**Fig 6:**
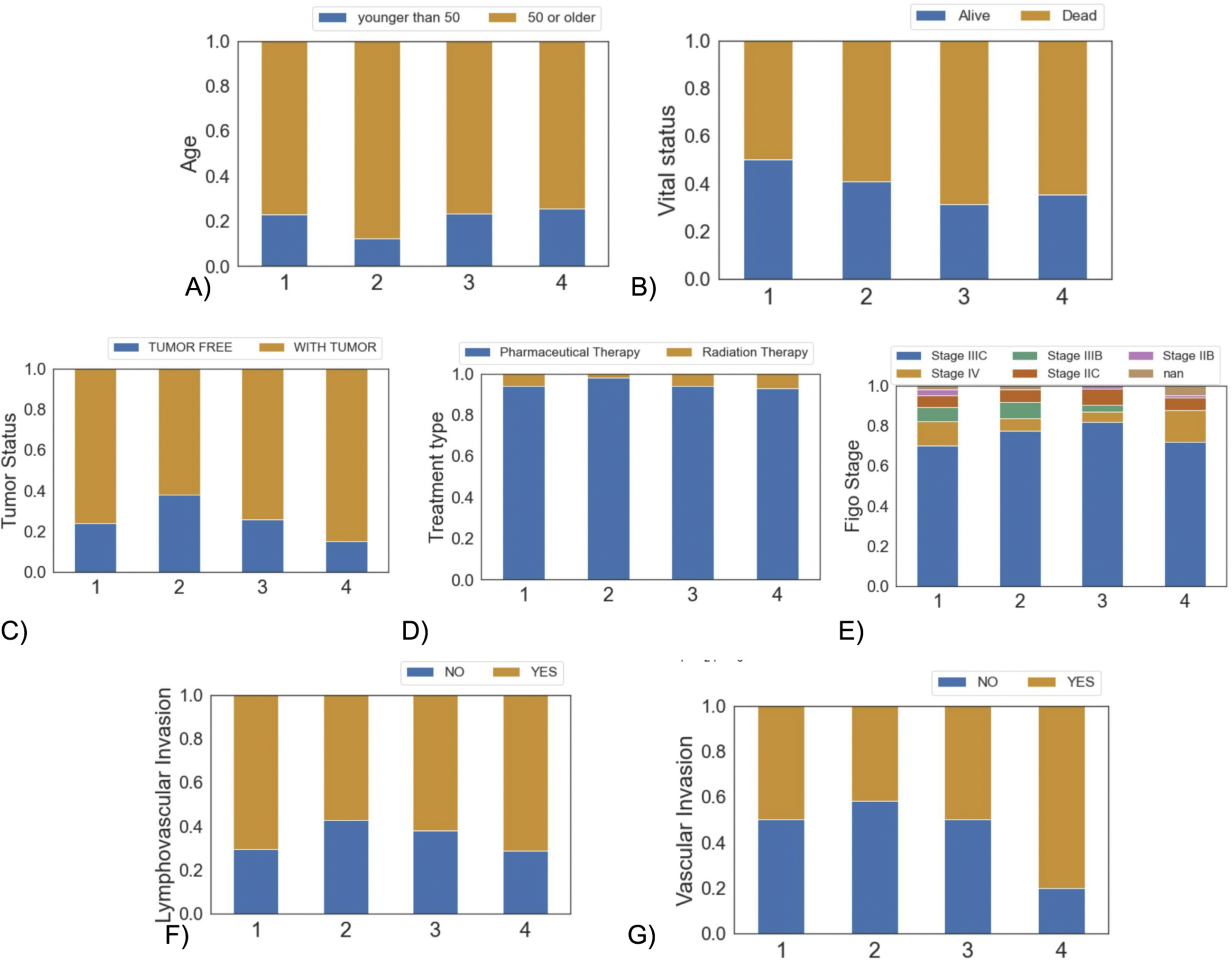
Barplot of clinical features per cluster. **(A)** Younger or older than 50, **(B)** vital status, **(C)** tumor status, **(D)** pharmaceutical vs radiation therapies, **(E)** Figo stage, **(F)** lymphovascular invasion, and **(G)** vascular invasion.

Next, we compared the clinical features of the clusters (Fig 6). Cluster 2 has the highest amount of patients aged 50 or older (Fig 6A), while other clusters are comparable. Cluster 3 has the highest rate of deceased patients among all clusters (Fig 6B). Across all clusters, the majority of patients present with residual tumors, with Cluster 4 having the highest percentage of patients with tumors while Cluster 2 has the lowest (Fig 6C). Pharmaceutical therapy is the dominant treatment method compared to radiation therapy across all clusters, with cluster 2 having the highest proportion of patients receiving this treatment type compared to radiation therapy (Fig 6D). Regarding tumor stage, most patients across all clusters are diagnosed with Figo stage IIIC. Notably, Cluster 4 contains the highest percentage of patients with stage IV cancer (Fig 6E). Furthermore, Cluster 4 also shows the greatest prevalence of patients with vascular invasion and lymphovascular invasion (Fig 6F and 6G). Supplemental Figure 25A-C in S1 File show nonsignificant clinical differences among clusters.

**Fig 7:**
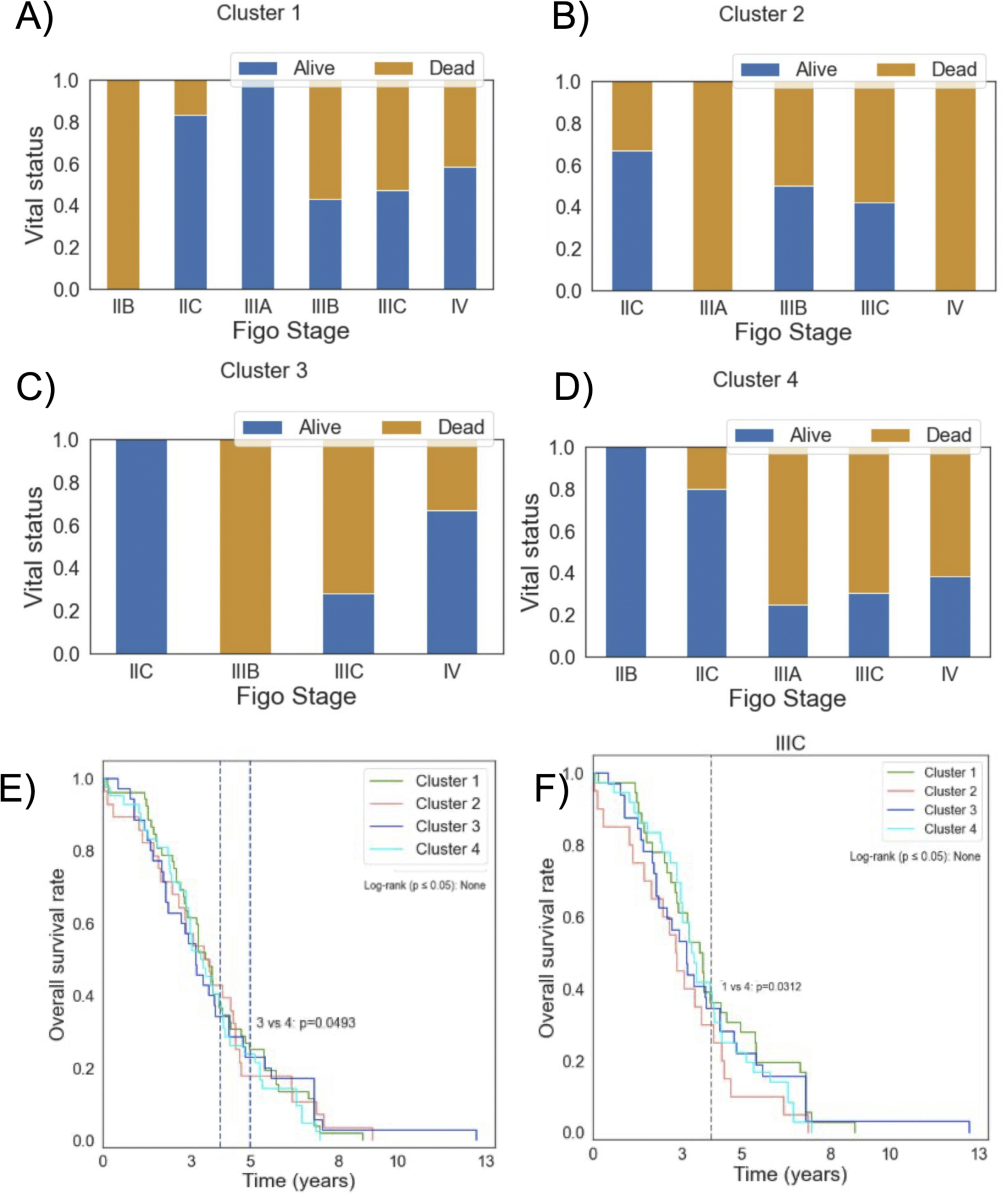
Kaplan-Meier survival analysis and stage-wise vital status. Sub-plots A-D show barplots of vital status per stage for clusters 1-4, respectively. Sub-plot (E) shows overall survival across 4 clusters. Sub-plot (F) shows survival curves for patients in stage IIIC across 4 clusters.

When investigating the Kaplan-Meier survival analysis for all patients across the four clusters, we did not observe any significant differences (Fig 7E). However, when we conducted a time-dependent analysis, we noticed a significant difference between clusters 3 and 4 after 5 years. (Supplemental Figure 26 A-B in S1 File).

Fig 7 (A-D) shows the difference in surviving patients by Figo stage for 4 clusters. No significant difference was found among all clusters (p-value> 0.05). Given that most diagnosed ovarian cancer cases are stage IIIC, according to Table 1, we conducted a stage-wise survival analysis for patients with stage IIIC ovarian cancer. A significant difference was found between clusters 1 and 4 after 4 years (p-value = 0.03) (Fig 7F). These results highlight the importance of pro-tumor macrophages (M2) and the activation mechanisms orchestrated by CD4 + T cells, given that the cluster with the poorest prognosis (cluster 4) is relatively enriched with these cell types. Additionally, the time-dependent survival differences may indicate a delay in the effectiveness of the immune response for patients. Survival analysis for stages III and IV, as well as survival of patients based on stage are in supplemental Figures 26 C-F in S1 File.

## Discussion

The tumor microenvironment has gained significant attention in cancer studies in recent years, both for understanding tumor biology and treatment strategies [4]. Ovarian cancer is known to be immunologically cold, which has made treatment more challenging than other cancer types [6]. Because the anti- or pro-tumor attribute of the immune landscape within the microenvironment depends on the balance of infiltrating immune systems, not their presence alone, we were curious whether their composition, ratio, subtypes, or polarization can reveal valuable prognostic insights or treatment options.

Although high-resolution techniques such as single-cell or spatial transcriptomics provide the most detailed view of the tumor microenvironment, bulk RNA-seq remains far more widely available across large cohorts such as TCGA. Computational deconvolution tools like CIBERSORTx enable the estimation of immune frequencies from widely available bulk RNA-seq data, providing a practical strategy for large-scale immune characterization. Prior studies have shown that bulk deconvolution can reliably recover the major cellular components of solid tumors when compared against tumor-derived single-cell references [31], and CIBERSORTx itself has been validated as a robust and scalable approach for inferring relative immune composition from bulk data [23]. Importantly, in this study, we benchmarked our inferred ranking of major immune populations against the cellular hierarchy reported in single-cell studies such as Schelker et al. [31], whose ovarian cancer immune reference profiles were derived from ascitic fluid samples rather than primary tumor tissue. Although ascitic fluid and primary tumor tissue represent distinct tumor microenvironments with potentially different immune functional states, the consistency in immune cell composition hierarchy across these datasets supports the immune patterns resulting from our deconvolution estimates. However, bulk deconvolution cannot resolve rare or transitional cell states, lacks spatial context, and depends on the quality of the signature matrix; therefore, our findings should be interpreted as reflecting relative differences in immune composition rather than precise cell quantification. Furthermore, our comparison with ascitic fluid-derived single-cell data serves to validate overall immune composition patterns rather than to imply functional equivalence between immune cells residing in ascitic fluid and those in primary tumor tissue.

Our results show that immune ratios, in particular CD8/Treg and CD8/CD4, were associated with better survival outcomes. However, the absolute infiltration levels of CD8, CD4, and Tregs, nor the overall immune score, were not prognostic. It has been shown that a subset of CD4 + cells expressing CTLA-4 and FOXP3 (Tregs) actively suppresses the immune response [49,50].

Moreover, Sato et al. in 2005 showed that groups with high CD8 + /CD4 + T cell ratios had higher survival than those with lower ratios, suggesting that effector–regulator balance mediated by CD4 + T cells influences the beneficial effects of CD8 + cells in the tumor microenvironment [15]. Consistent with this concept, prior studies in independent cohorts have reported that a high CD8 + /Treg ratio is associated with improved responses to immune checkpoint inhibitors, including pembrolizumab, in high-grade serous ovarian carcinoma [15,49,51]. However, immunotherapy response was not evaluated in our cohort, and our findings specifically demonstrate the prognostic significance of immune cell ratios in relation to survival outcomes. These observations support therapeutic strategies that enhance CD8 + cytotoxic activity while restraining Treg-driven suppression, rather than broadly increasing immune infiltration.

Macrophages have been the subject of many studies recently, and it has been established that they can act in favor of or against a patient’s survival depending on their polarization status [52]. In our cohort, we found that higher infiltration of pro-tumor macrophages (M2) was significantly associated with worse overall survival (Fig 3C), increased vascular invasion (Fig 2C), and persistent tumor status at follow-up (Fig 2E), demonstrating the prognostic importance of M2 polarization across multiple clinical endpoints. These behaviors are aligned with other findings in the field [30,53]. Interestingly, our analysis revealed that higher naive (M0) macrophages were associated with improved survival outcomes (Fig 3A and 3B), with no implication of prognostic values for anti-tumor (M1) macrophages. While puzzling, this can have crucial implications. The ovarian tumor microenvironment is exceptionally immunosuppressive [6]. This will lead to pathways that favor M2 polarizations. Thus, a larger apparent pool of M0 macrophages can be an indication of a less immunosuppressive microenvironment, potentially contributing to improved immune surveillance and survival outcomes observed in our cohort.

Although neutrophil infiltration was uncommon overall, elevated neutrophil levels were associated with poor survival. Recent evidence has established that neutrophils paralyze the T cell immune response through mechanisms distinct from classic checkpoint pathways [54]. Our results complement this mechanistic insight, elevating neutrophils from bystanders to active suppressors in ovarian cancer, and suggest that targeting them may reverse T-cell paralysis, leading to better prognosis.

We found significant associations between the infiltrating immune cells (i.e., CD8 + T cells, Tregs, M2 macrophages, neutrophils, and monocytes) and conditions that lead to poor prognosis, such as vascular and lymphovascular invasion. These conditions are representative of a more aggressive tumor (in a later stage) [55], and the response implies that the immune system has fully recognized the danger (i.e., a significant CD8 + recruitment). However, the same aggressive, inflamed microenvironment also recruits immunosuppressive partners (i.e., Tregs, M2 macrophages, neutrophils, and monocytes), which overwhelm the CD8 + anti-tumor response. As lymphovascular and vascular invasion are established hallmarks of aggressive disease, their association with this unbalanced immune profile facilitates an immunosuppressive environment, resulting in a “colder tumor” less likely to respond to therapy. This interpretation is further supported by our finding that immune cell ratios, particularly CD8/Treg and CD8/CD4, were more strongly associated with survival outcomes than absolute immune cell infiltration levels, highlighting the importance of effector–regulator balance rather than overall immune abundance. Prior studies have similarly reported that higher CD8/Treg ratios are associated with improved immune-mediated tumor control and response to immune checkpoint inhibitors in independent cohorts; however, immunotherapy response was not evaluated in our dataset. These findings emphasize that an imbalance favoring immunosuppressive populations can negate the beneficial effects of CD8 + T cell recruitment, contributing to worse clinical outcomes.

Our unsupervised clustering stratified the patients into four distinct immune profiles, primarily differentiated by M0 vs. M2 macrophages, with contributions from CD4+ and plasma cells. The overall survival did not differ across clusters at baseline. However, time-dependent divergence emerged after 5 years. Given that most patients belonged to FIGO stage IIIC, limiting the survival analysis to this group yielded more substantial divergence after 4 years. As mentioned, later stages are accompanied by conditions such as lymphovascular and vascular invasion, and these conditions contribute to a colder tumor microenvironment. Notably, the cluster that had the most M2 macrophages and CD4 + T cells, and at the same time the lowest M0 macrophages, showed the worst outcomes. This cluster also demonstrated enrichment of regulatory and helper immune populations that are known to contribute to immunosuppressive signaling, providing a mechanistic explanation for the observed survival differences. This further confirms the importance of immunosuppression role players in ovarian cancer prognosis, specifically in later stages.

There are some limitations to consider when interpreting the results of this paper. First, we have utilized deconvoluted bulk RNA-seq data; hence, our analyses lack spatial resolution and cannot assign polarization states at the single-cell level. Second, the number of data points for most stages of OC is low compared to later stages (i.e., IIIC and IV). Therefore, the deductions are more generalizable for later stages rather than early ones. Third, survival stratification used median/quantile cutoffs, which, while common, are threshold-dependent. Finally, findings are derived from a single cohort (TCGA) and can benefit from further external validation.

Overall, our findings underscore the pivotal role of the immune landscape, particularly the prevalence of an immunosuppressive tumor microenvironment, in determining outcomes in ovarian cancer, one of the most immunologically cold malignancies. We identified specific immune cells, namely M2 macrophages and neutrophils, that may drive this immune-cold phenotype. Surprisingly, naïve (M0) macrophages were strongly associated with improved prognosis, whereas anti-tumor (M1) macrophages showed no such effect. This suggests that therapeutic strategies aimed at preserving M0 macrophages or guiding their polarization toward anti-tumor phenotypes may have some treatment benefits. In addition, immune cell ratios such as CD8/Treg and CD8/CD4 proved to be more informative than the absolute abundance of individual cell types, which were not prognostic. These ratios better capture the balance between effector and suppressive forces within the tumor microenvironment. Future studies integrating spatial immune profiling will be crucial to validate these findings and elucidate the mechanisms of immune suppression specific to ovarian cancer. Ultimately, incorporating immune ratios, neutrophil’s T cell paralyzing pathways, and macrophage polarization into clinical risk models could improve patient stratification and guide the design of more effective immunotherapies.

## Supporting information

S1 File Associations between immune cell populations and clinical features, survival analyses, and clustering results. Supplemental Figures 1–19 show associations between immune cell ratios/populations (CD8/CD4 ratio, CD8/Tregs ratio, M1/M2 ratio, Neutrophils, Eosinophils, T gamma delta cells, CD4 + T naive cells, Mast cells, Dendritic cells, Monocytes, CD8 + T cells, NK cells, Tregs, B cells, Plasma cells, M1 Macrophages, M0 Macrophages, M2 Macrophages, and CD4 + T cells) and clinical features including lymphovascular invasion, vascular invasion, treatment type, early- vs late-onset, vital status, and tumor status. Supplemental Figures 20–23 show Kaplan–Meier survival analyses based on immune cell ratios using median, upper quartile, and lower quartile cut-offs. Supplemental Figures 24–26 show immune composition clustering results, clinical features per cluster, and cluster-based survival analyses.
(DOCX)
