# Supplementary material for: Analysis of ovarian cancer immune cell profile identifies immunosuppressive states associated with adverse clinical attributes and survival times
Source: PLoS One. 2026 Apr 20;21(4):e0346746. doi: 10.1371/journal.pone.0346746 (PMC13094978; doi:10.1371/journal.pone.0346746)
Supplement: S1 File — Supplemental Figures 1–19 show associations between immune cell ratios/populations (CD8/CD4 ratio, CD8/Tregs ratio, M1/M2 ratio, Neutrophils, Eosinophils, T gamma delta cells, CD4 + T naive cells, Mast cells, Dendritic cells, Monocytes, CD8 + T cells, NK cells, Tregs, B cells, Plasma cells, M1 Macrophages, M0 Macrophages, M2 Macrophages, and CD4 + T cells) and clinical features including lymphovascular invasion, vascular invasion, treatment type, early- vs late-onset, vital status, and tumor status. Supplemental Figures 20–23 show Kaplan–Meier survival analyses based on immune cell ratios using median, upper quartile, and lower quartile cut-offs. Supplemental Figures 24–26 show immune composition clustering results, clinical features per cluster, and cluster-based survival analyses. (DOCX) [file pone.0346746.s001.docx]

A)
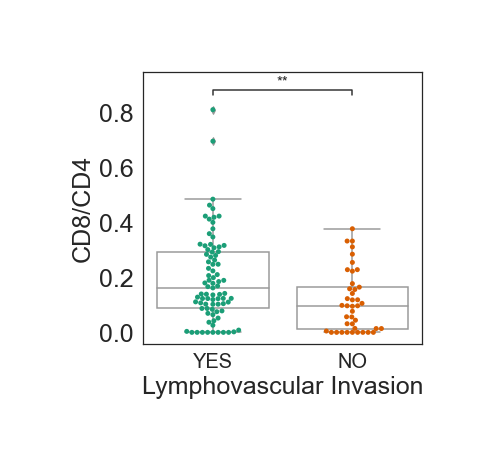
B)
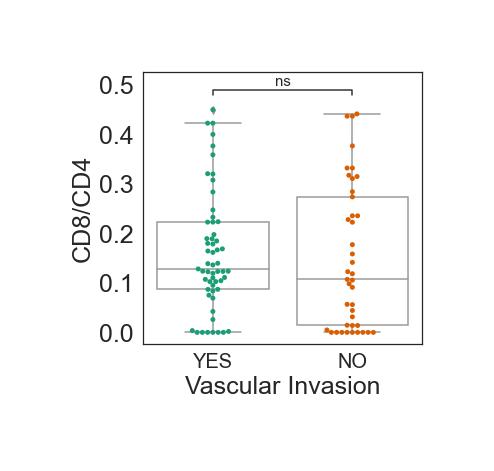
C)
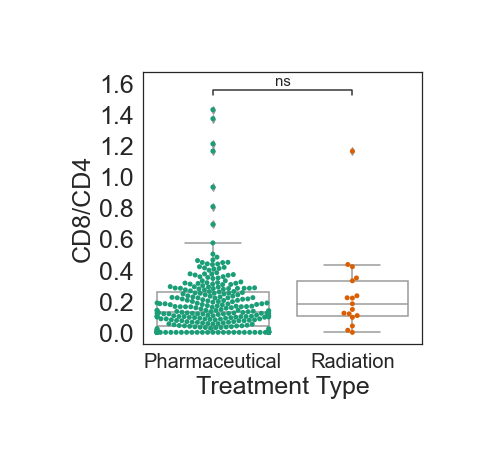


D)
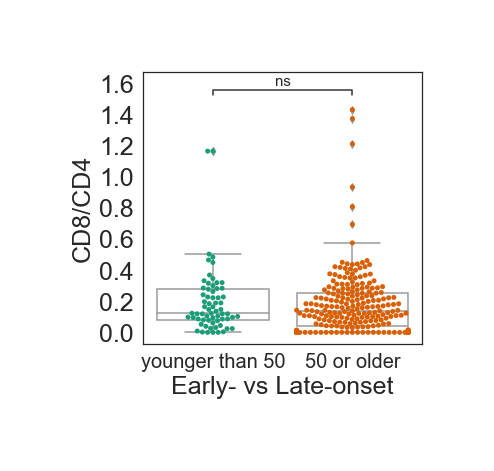
E)
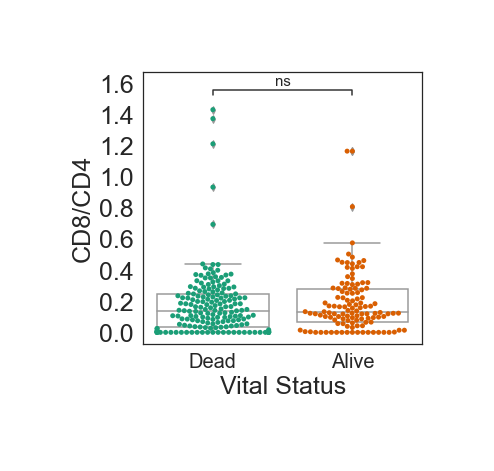
F)
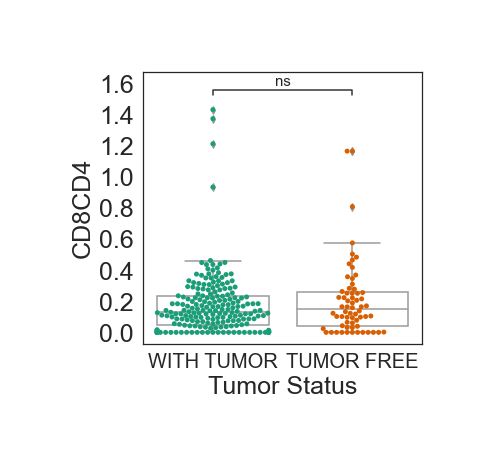


Supplemental Figure 1: Associations Between CD8/CD4 ratio and Clinical Features. A) Lymphovascular invasion. B) Vascular Invasion. C) Treatment Type. D) Early- vs Late-onset. E) Vital status. F) Tumor status.

A)
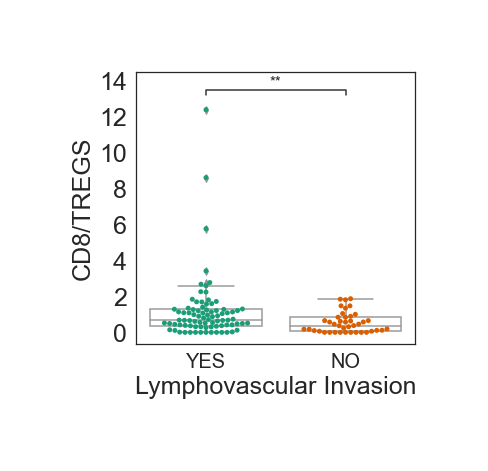
B)
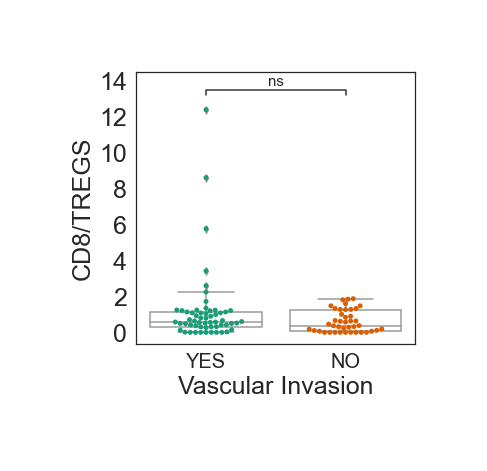
C)
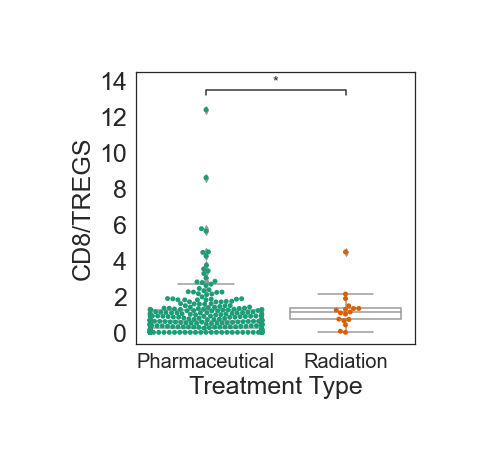


D)
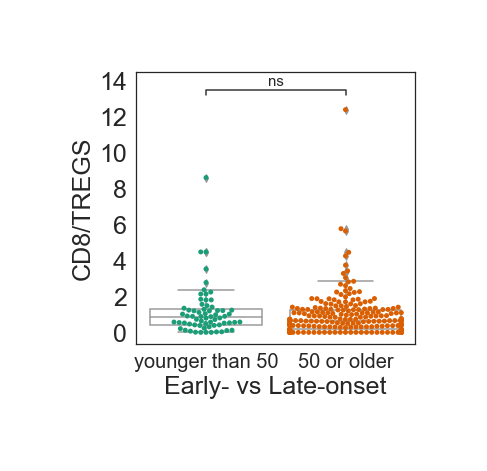
E)
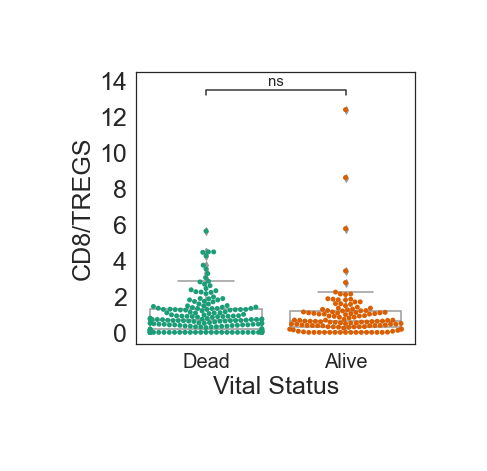
F)
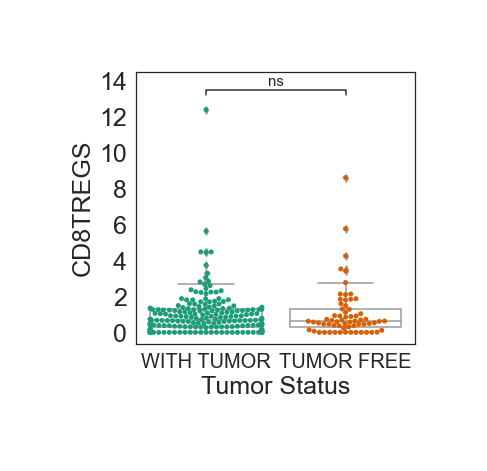


Supplemental Figure 2: Associations Between CD8/Tregs ratio and Clinical Features. A) Lymphovascular invasion. B) Vascular Invasion. C) Treatment Type. D) Early- vs Late-onset. E) Vital status. F) Tumor status.

A)
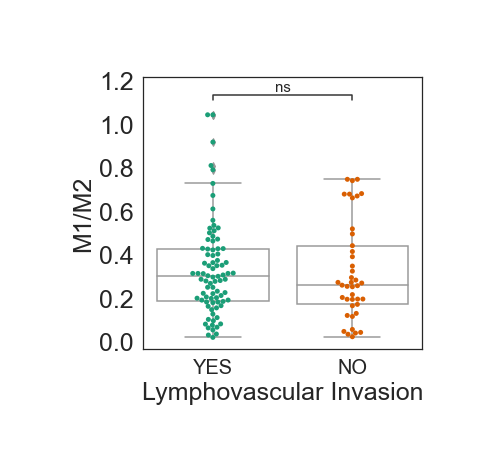
B)
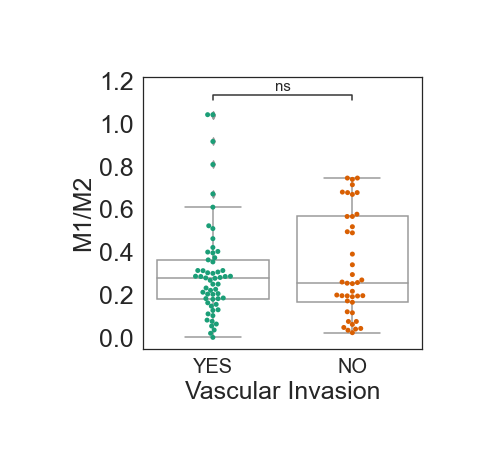
C)
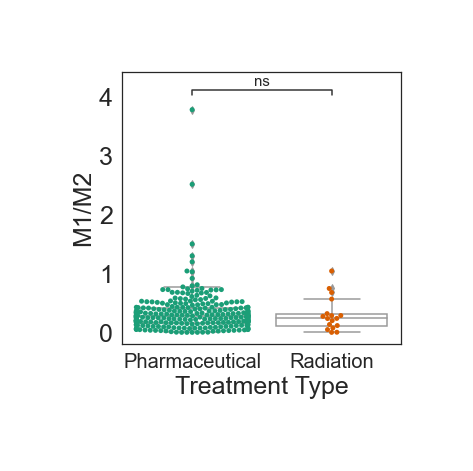


D)
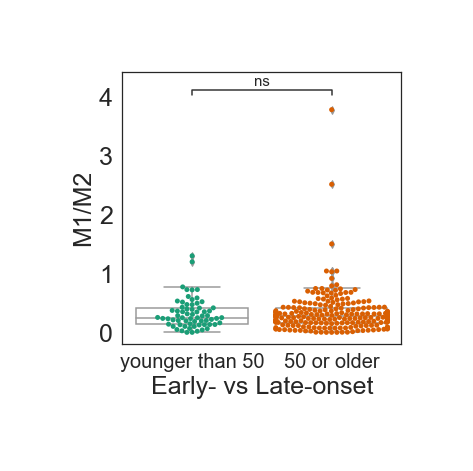
E)
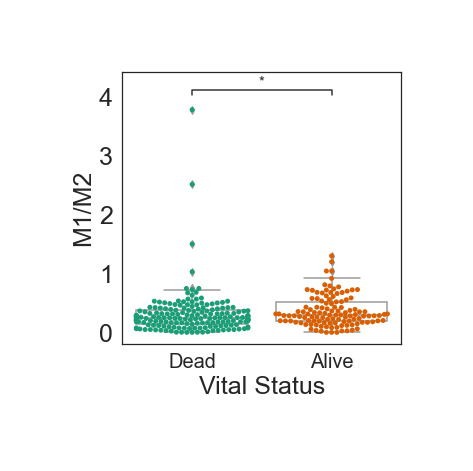
F)
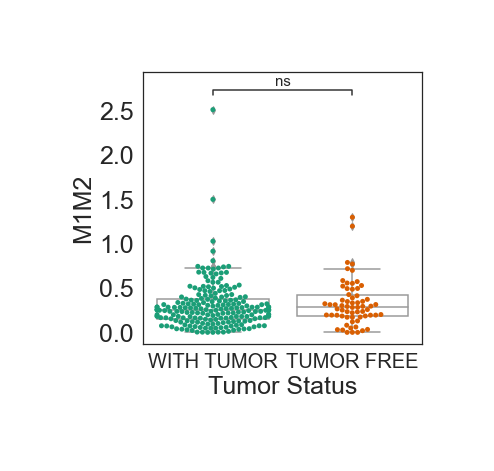


Supplemental Figure 3: Associations Between M1/M2 ratio and Clinical Features. A) Lymphovascular invasion. B) Vascular Invasion. C) Treatment Type. D) Early- vs Late-onset. E) Vital status. F) Tumor status.

A)
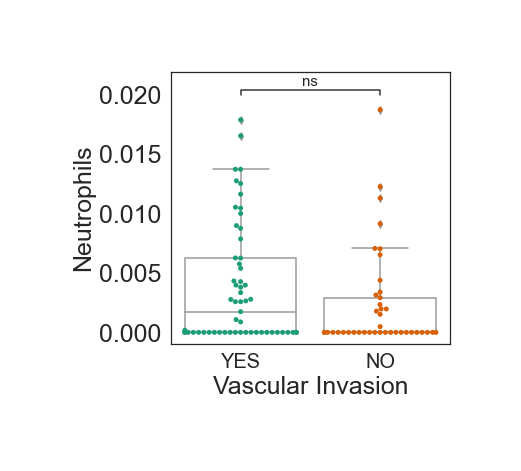
B)
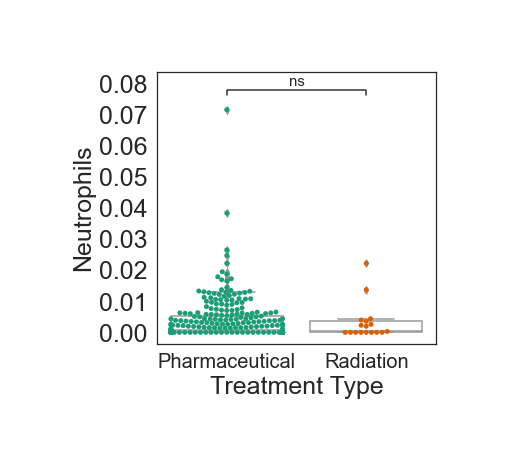
C)
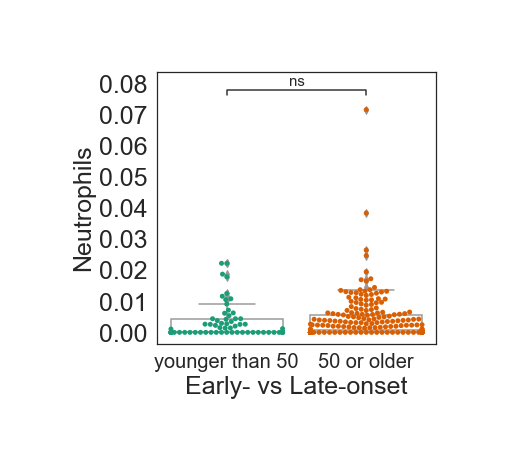


D)
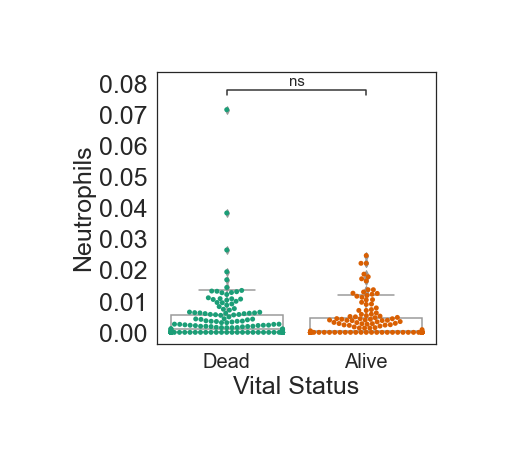
E)
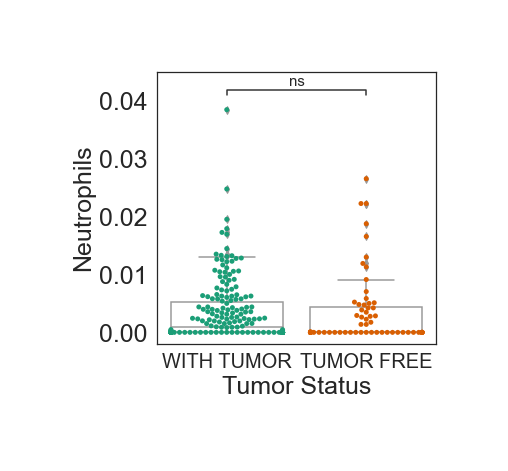


Supplemental Figure 4: Associations Between Neutrophils and Clinical Features. A) Vascular Invasion. B) Treatment Type. C) Early- vs Late-onset. D) Vital status. E) Tumor status.

A)
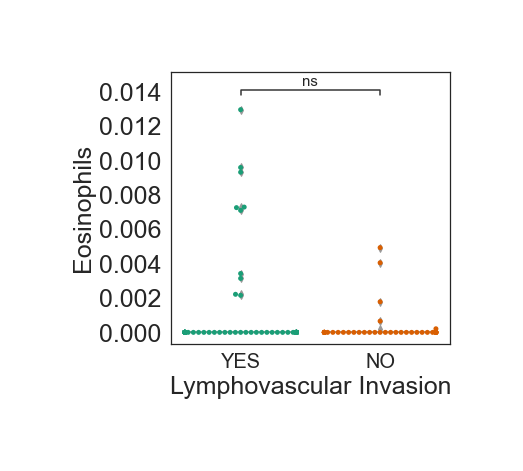
B)
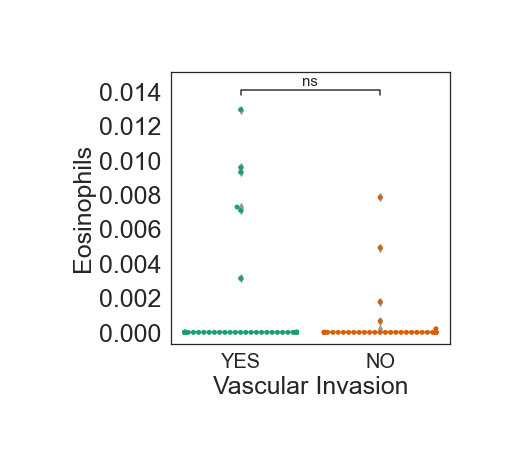
C)
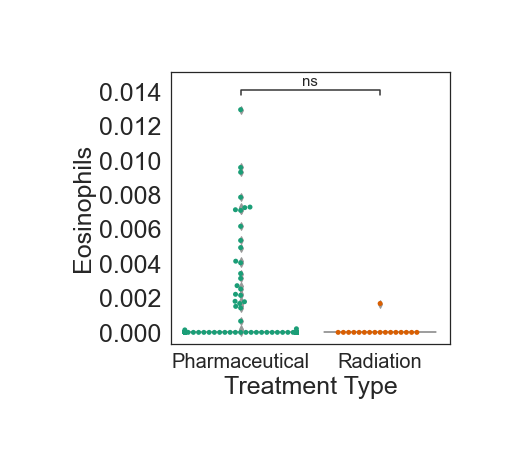


D)
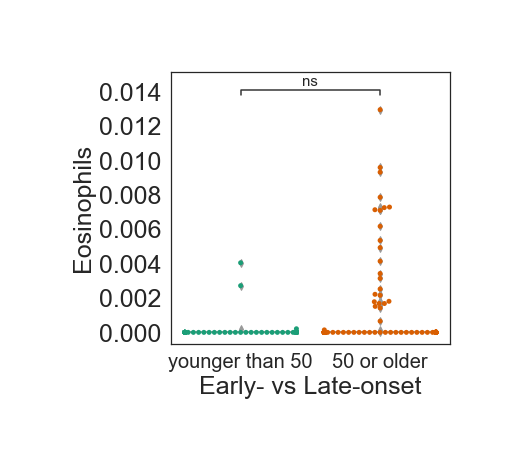
E)
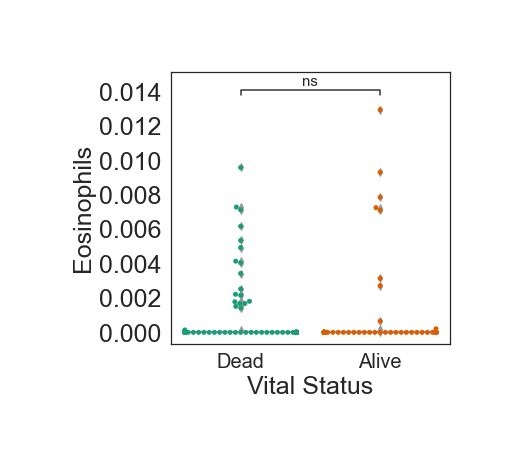
F)
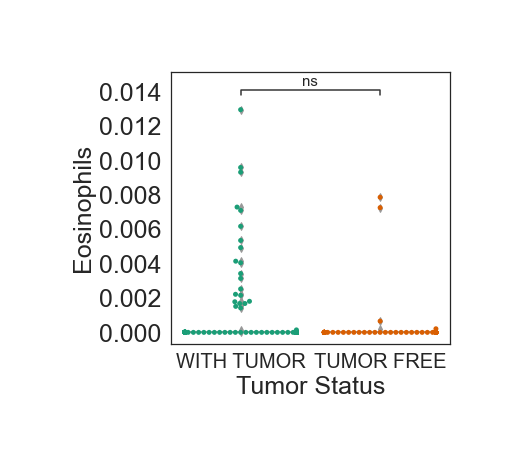


Supplemental Figure 5: Associations Between Eosinophils and Clinical Features. A) Lymphovascular invasion. B) Vascular Invasion. C) Treatment Type. D) Early- vs Late-onset. E) Vital status. F) Tumor status.

A)
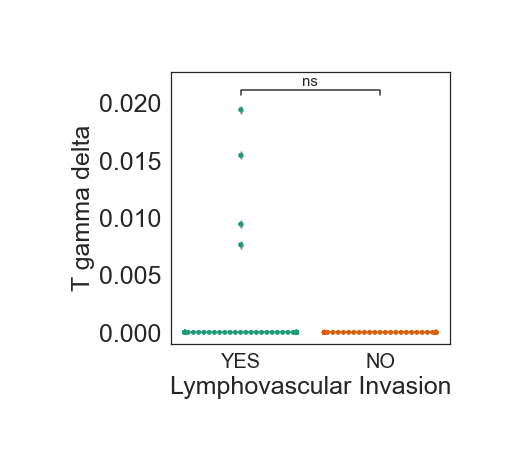
B)
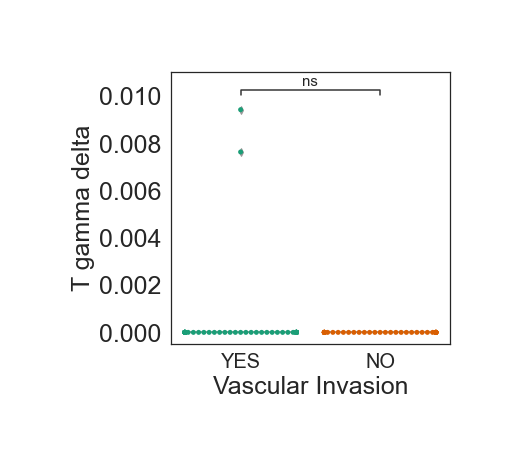
C)
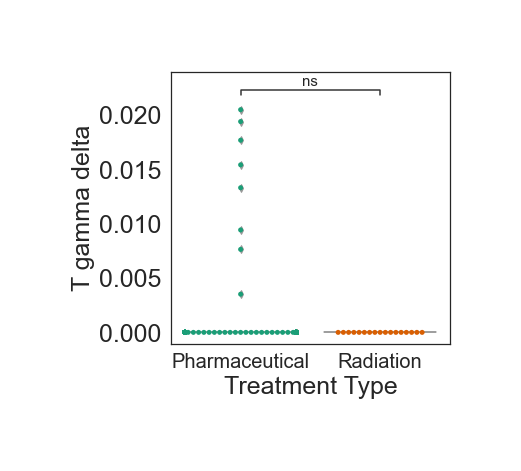


D)
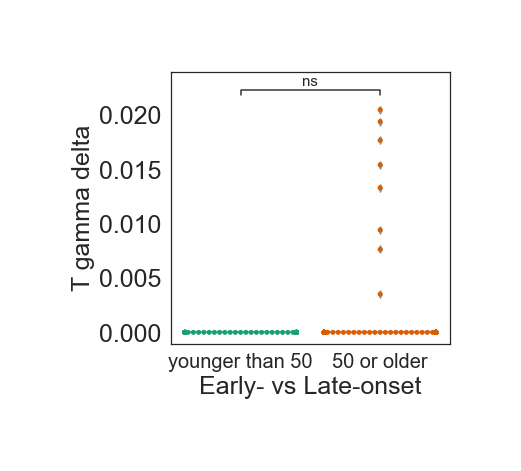
E)
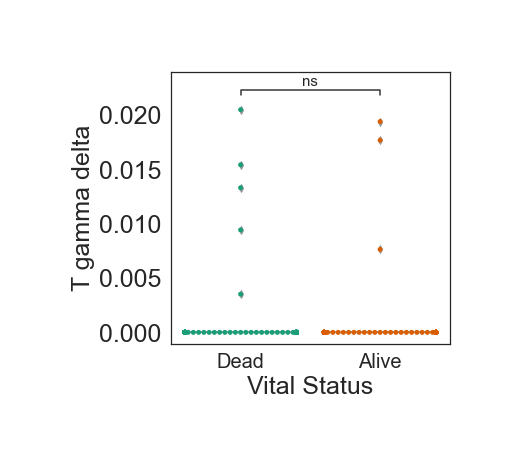
F)
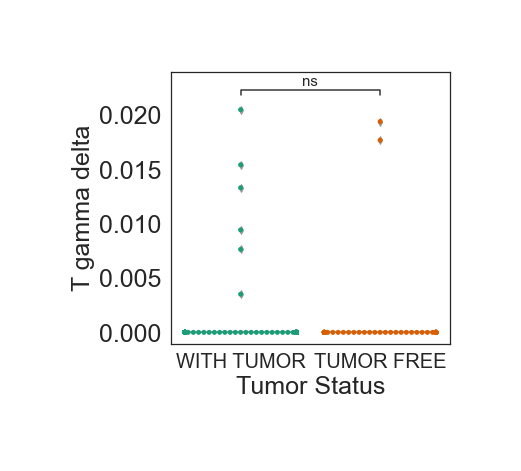


Supplemental Figure 6: Associations Between T gamma delta cells and Clinical Features. A) Lymphovascular invasion. B) Vascular Invasion. C) Treatment Type. D) Early- vs Late-onset. E) Vital status. F) Tumor status.

A)
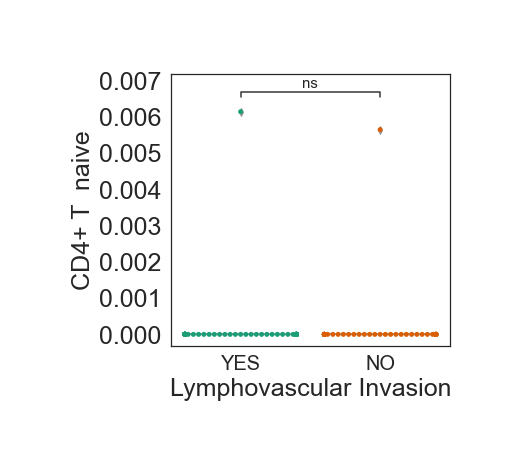
B)
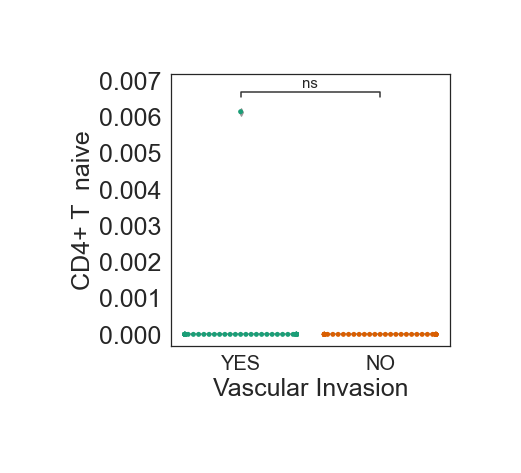
C)
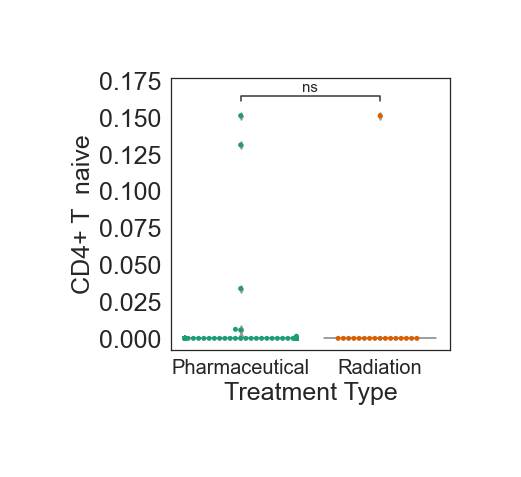


D)
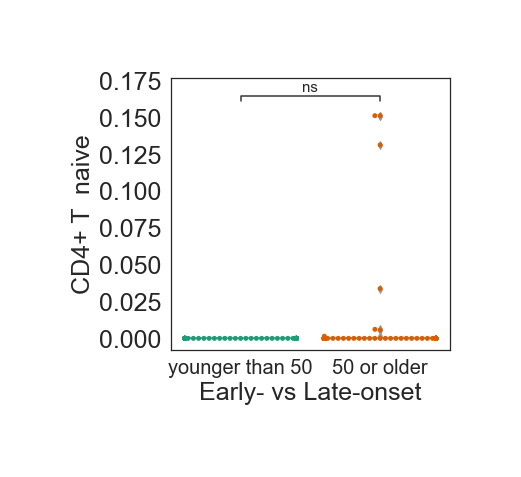
E)
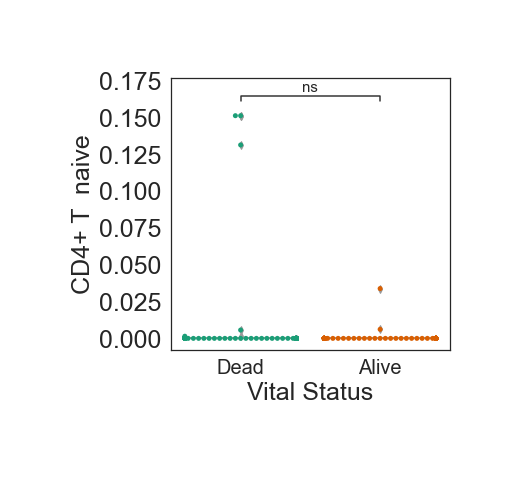
F)
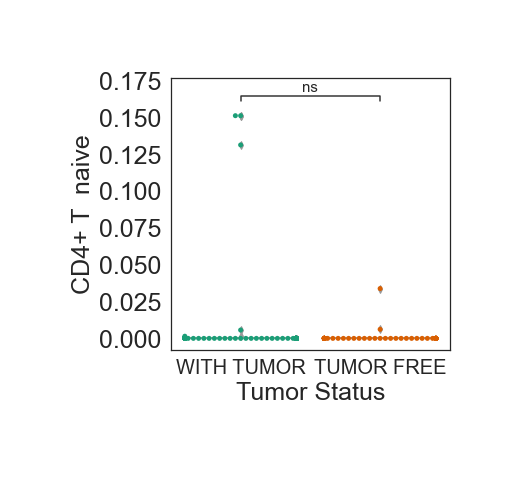


Supplemental Figure 7: Associations Between CD4+ T naive cells and Clinical Features. A) Lymphovascular invasion. B) Vascular Invasion. C) Treatment Type. D) Early- vs Late-onset. E) Vital status. F) Tumor status.

A)
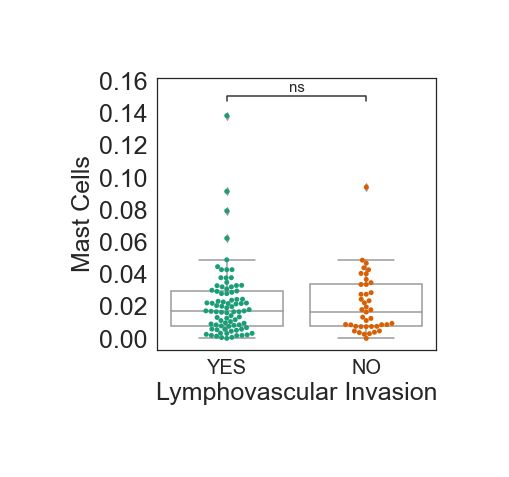
B)
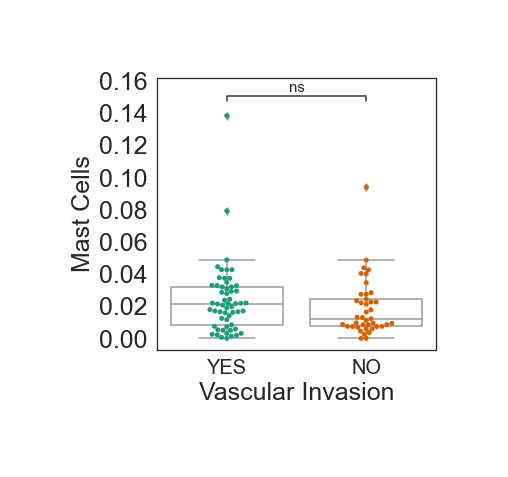
C)
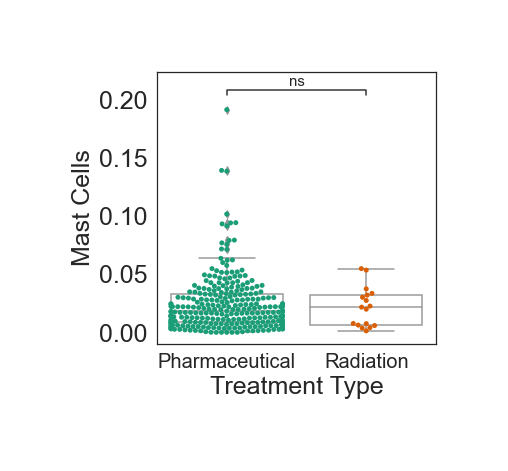


D)
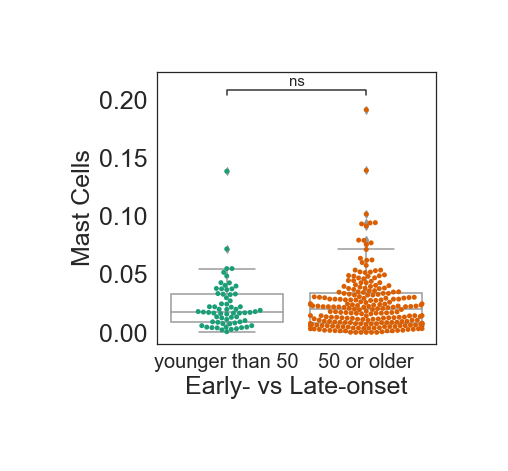
E)
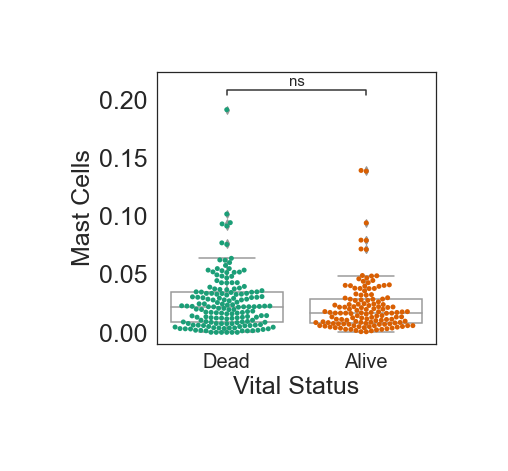
F)
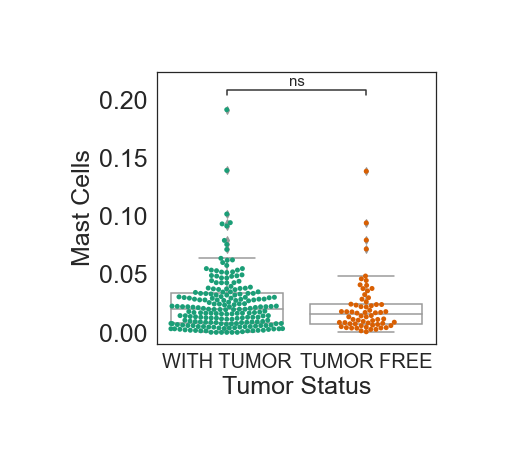


Supplemental Figure 8: Associations Between Mast Cells and Clinical Features. A) Lymphovascular invasion. B) Vascular Invasion. C) Treatment Type. D) Early- vs Late-onset. E) Vital status. F) Tumor status.

A)
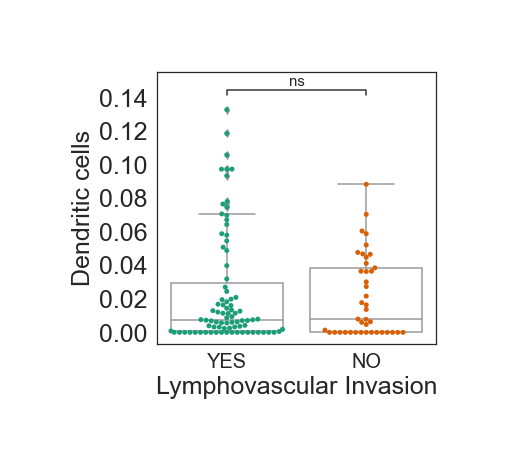
B)
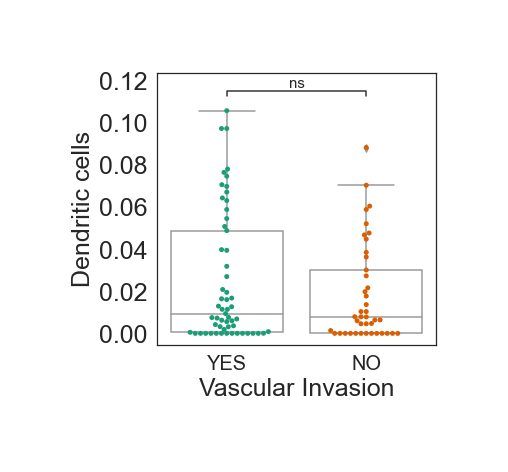
C)
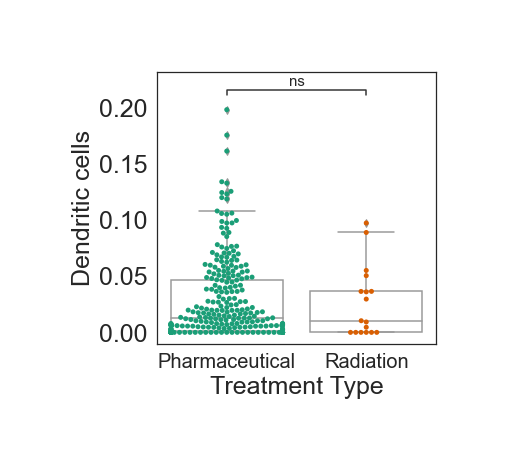


D)
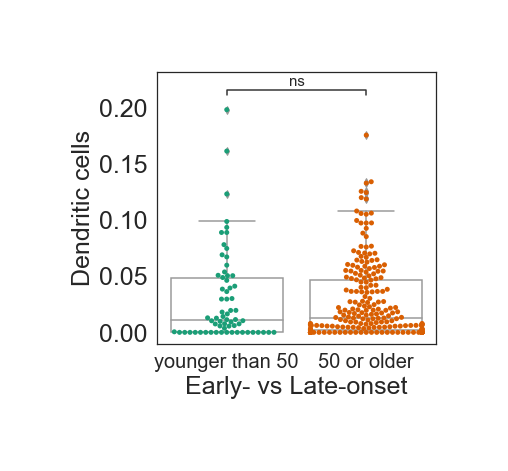
E)
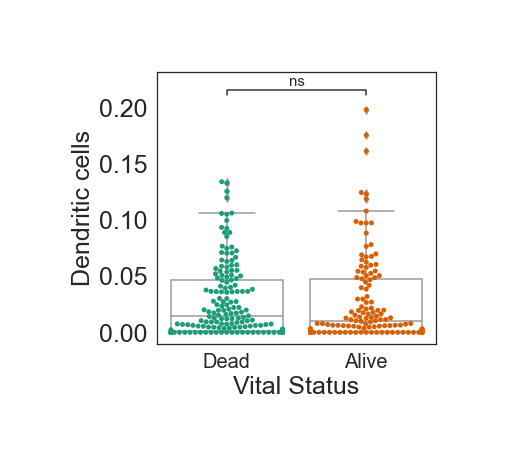
F)
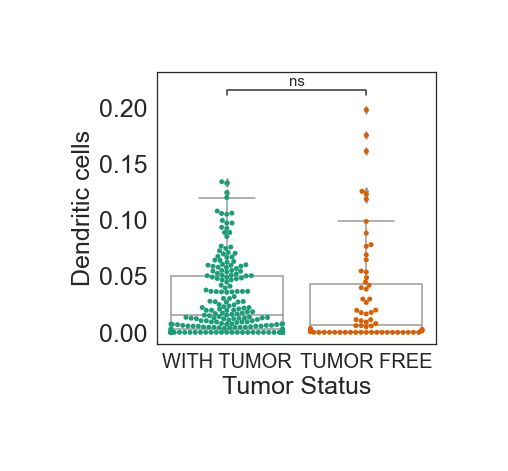


Supplemental Figure 9: Associations Between Dendritic Cells and Clinical Features. A) Lymphovascular invasion. B) Vascular Invasion. C) Treatment Type. D) Early- vs Late-onset. E) Vital status. F) Tumor status.

A)
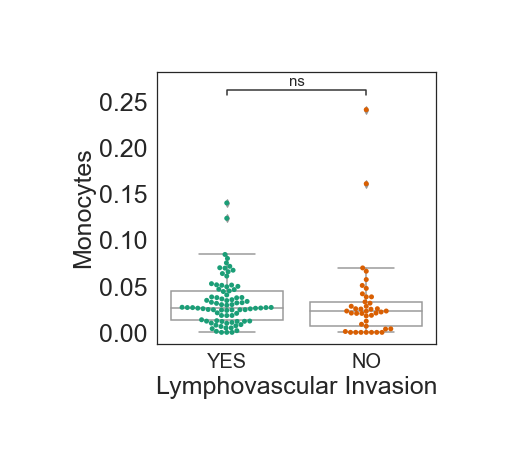
B)
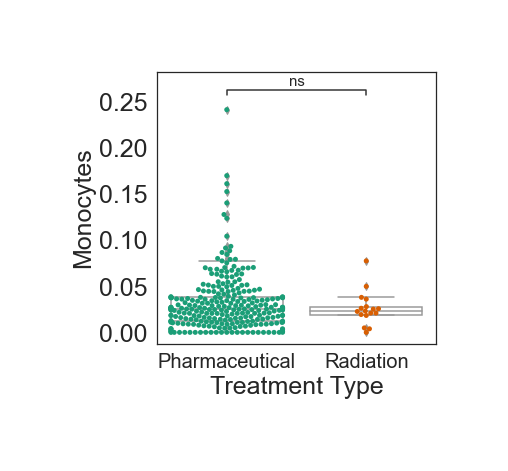
C)
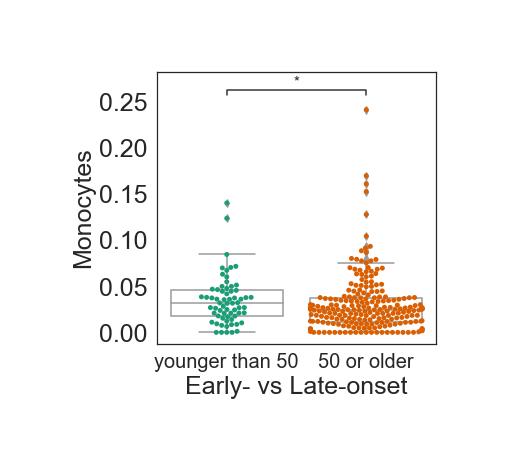


D)
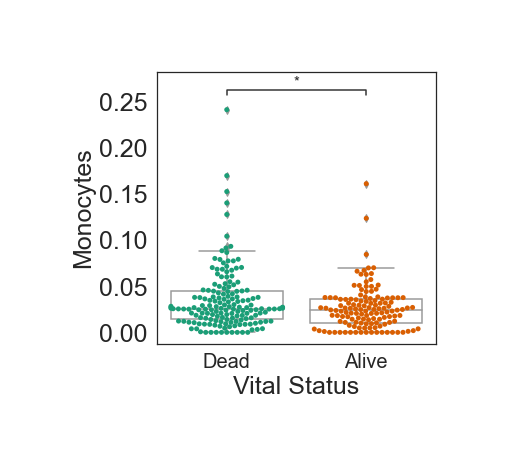
E)
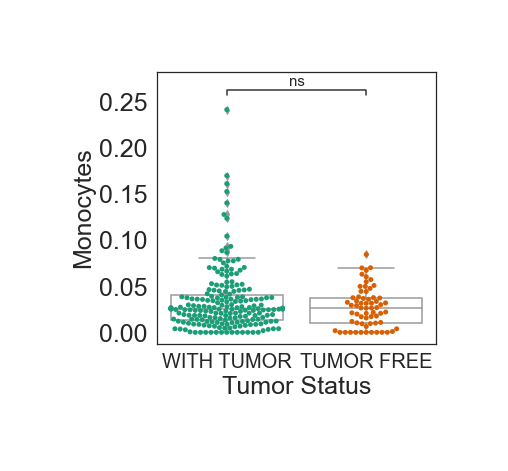


Supplemental Figure 10: Associations Between Monocytes and Clinical Features. A) Lymphovascular invasion. B) Treatment Type. C) Early- vs Late-onset. D) Vital status. E) Tumor status.

A)
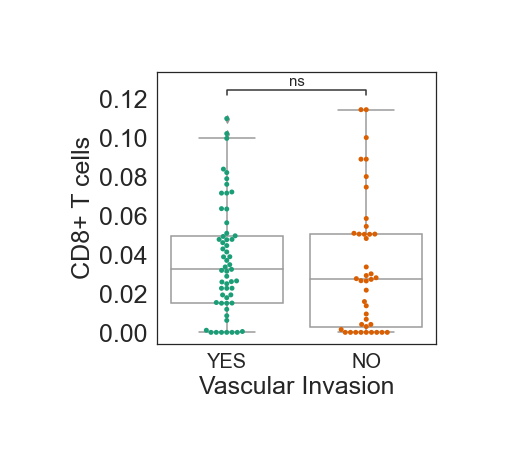
B)
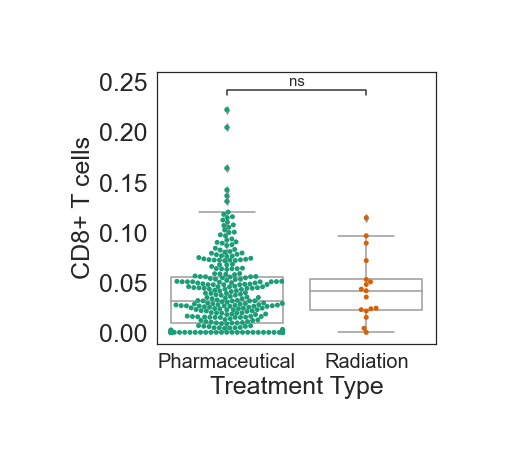
C)
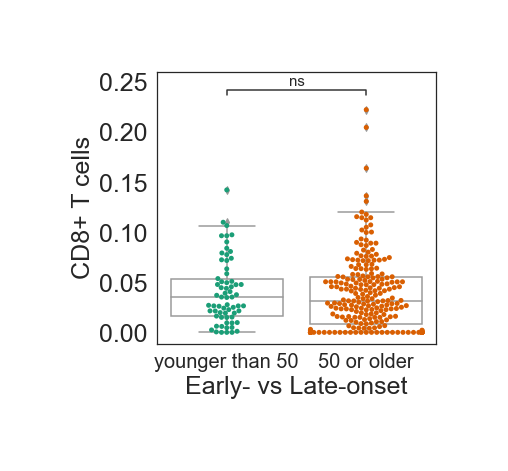


D)
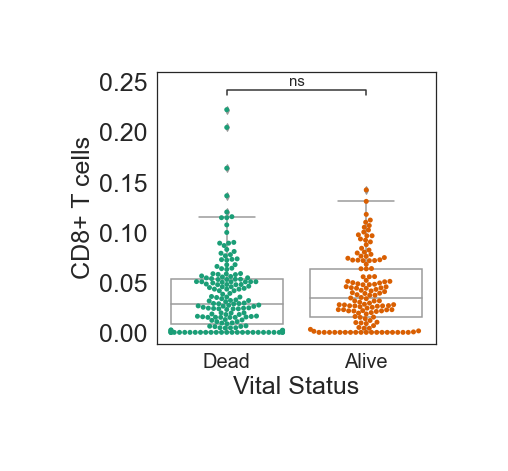
E)
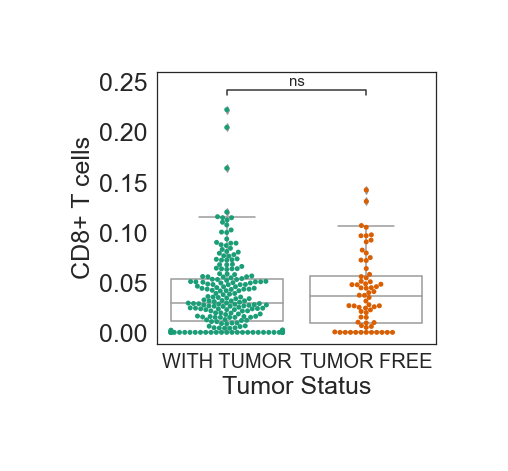


Supplemental Figure 11: Associations Between CD8+ T Cells and Clinical Features. A) Vascular Invasion. B) Treatment Type. C) Early- vs Late-onset. D) Vital status. E) Tumor status.

A)
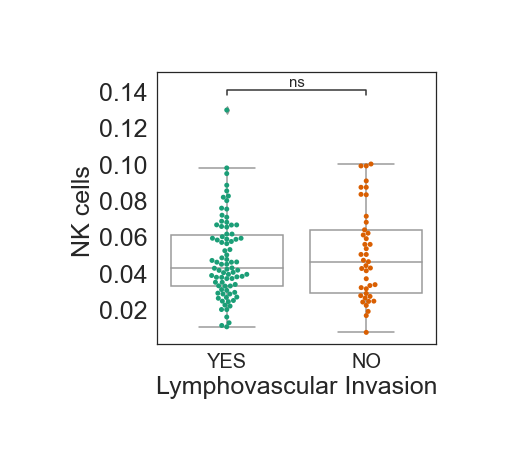
B)
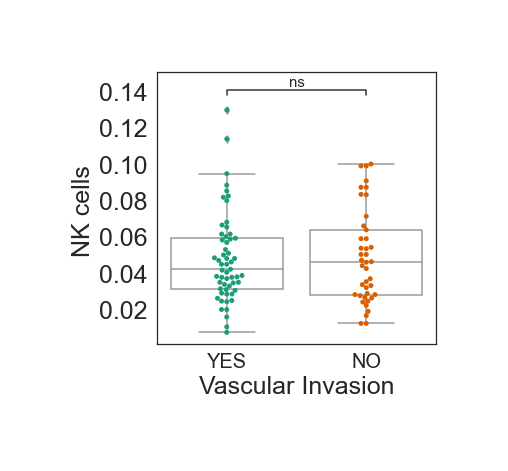
C)
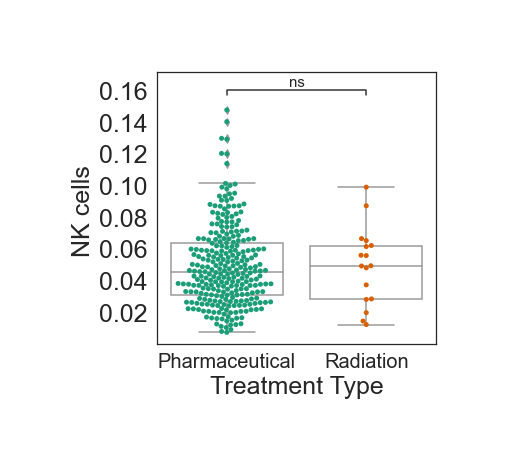


D)
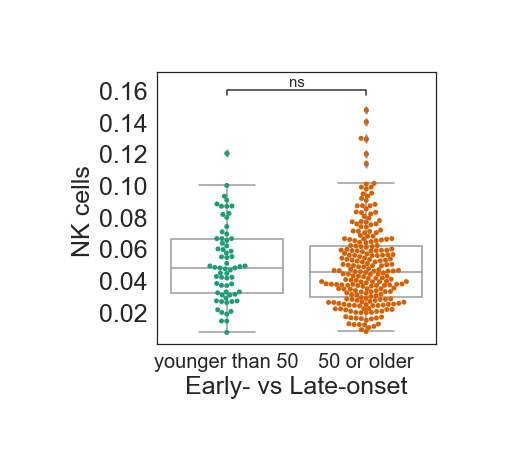
E)
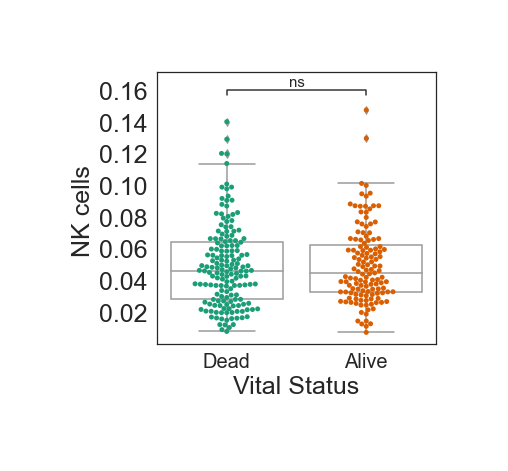
F)
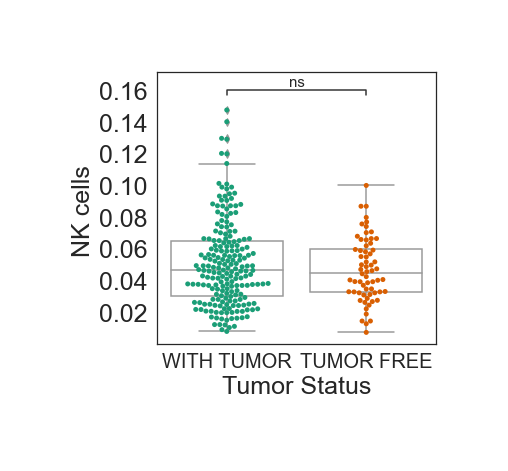


Supplemental Figure 12: Associations Between NK Cells and Clinical Features. A) Lymphovascular invasion. B) Vascular Invasion. C) Treatment Type. D) Early- vs Late-onset. E) Vital status. F) Tumor status.

A)
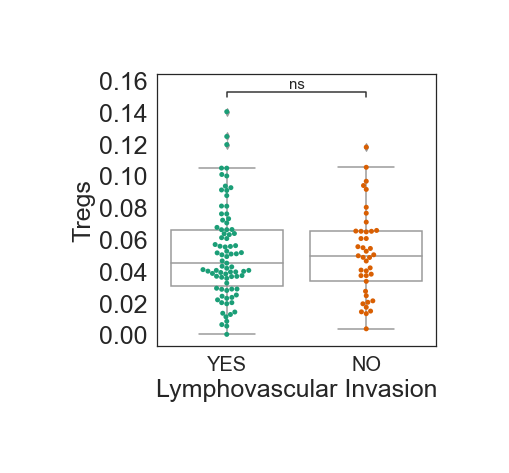
B)
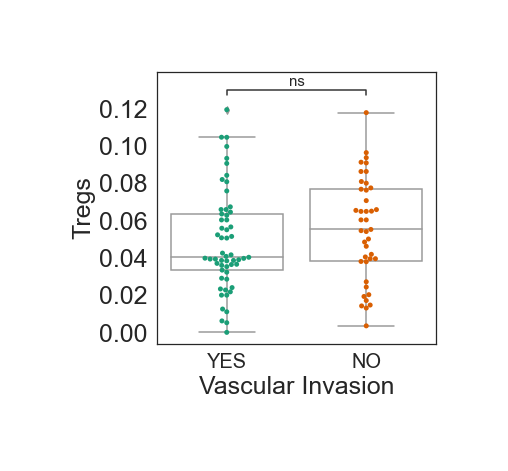
C)
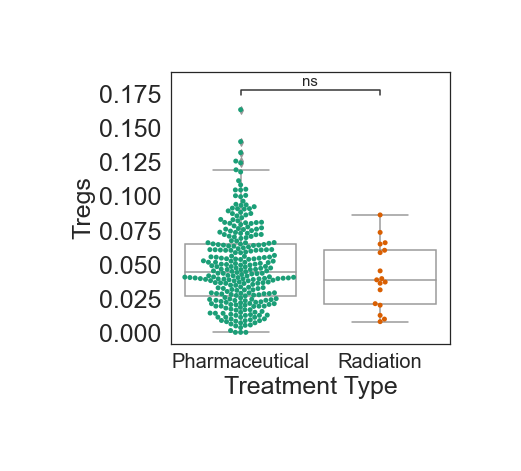


D)
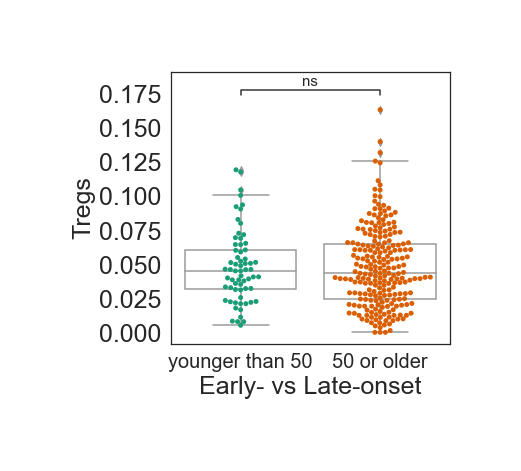
E)
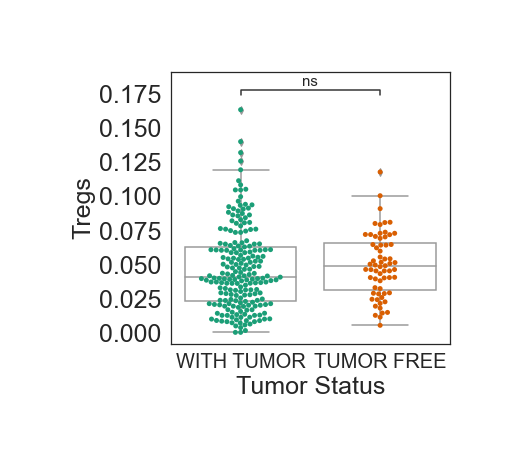


Supplemental Figure 13: Associations Between Tregs and Clinical Features. A) Lymphovascular invasion. B) Vascular Invasion. C) Treatment Type. D) Early- vs Late-onset. E) Tumor status.

A)
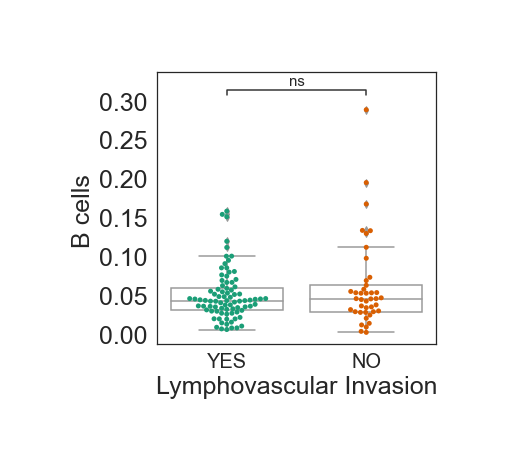
B)
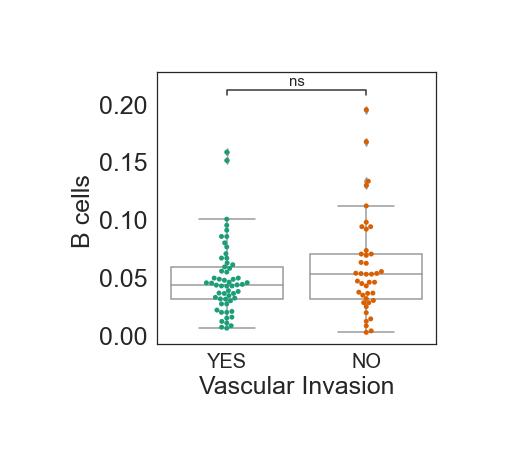
C)
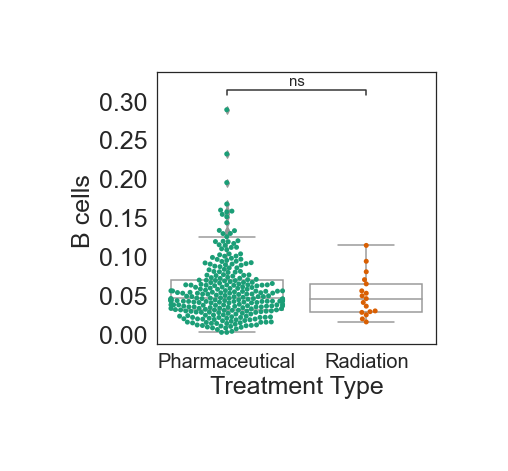


D)
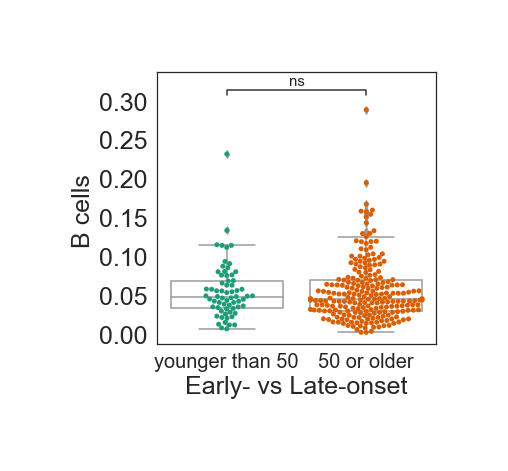
E)
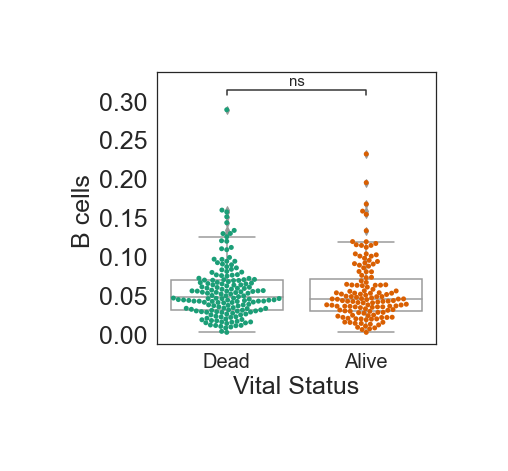


Supplemental Figure 14: Associations Between B Cells and Clinical Features. A) Lymphovascular invasion. B) Vascular Invasion. C) Treatment Type. D) Early- vs Late-onset. E) Vital status.

A)
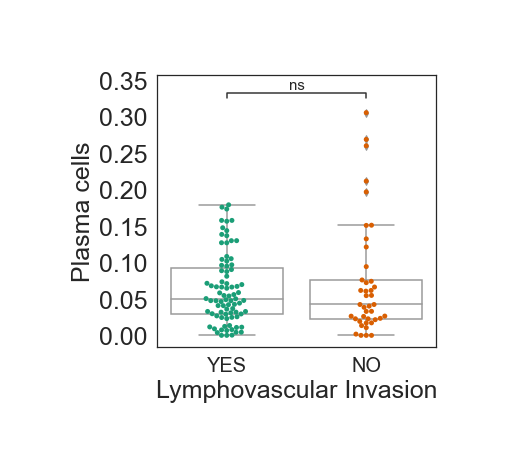
B)
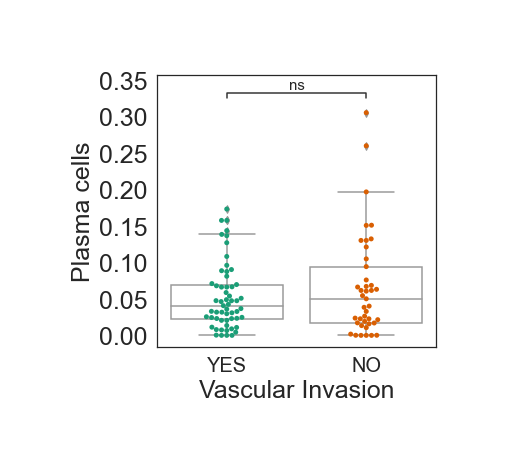
C)
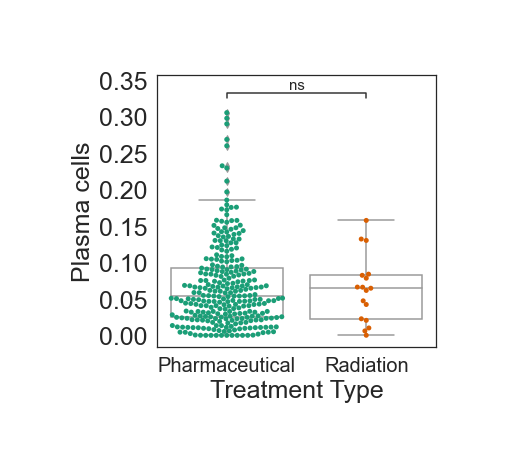


D)
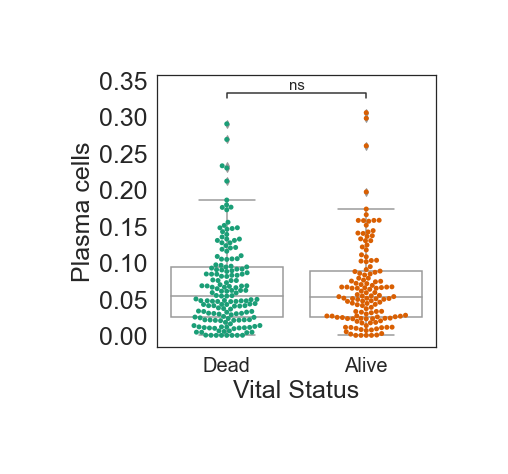
E)
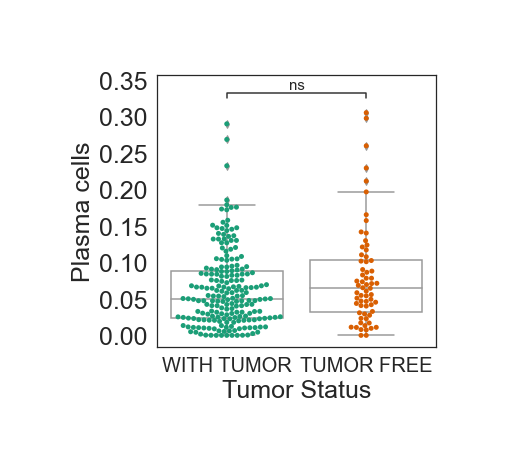


Supplemental Figure 15: Associations Between Dendritic Cells and Clinical Features. A) Lymphovascular invasion. B) Vascular Invasion. C) Treatment Type. D) Vital status. E) Tumor status.

A)
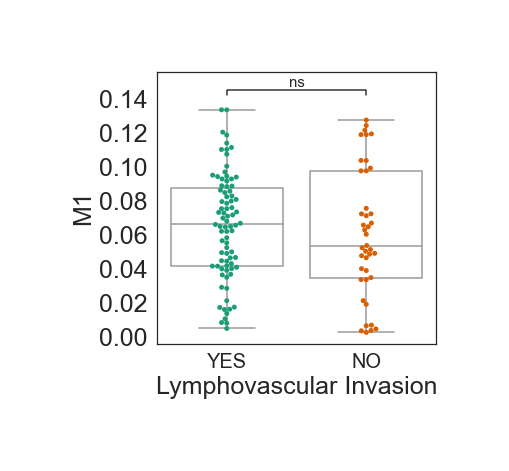
B)
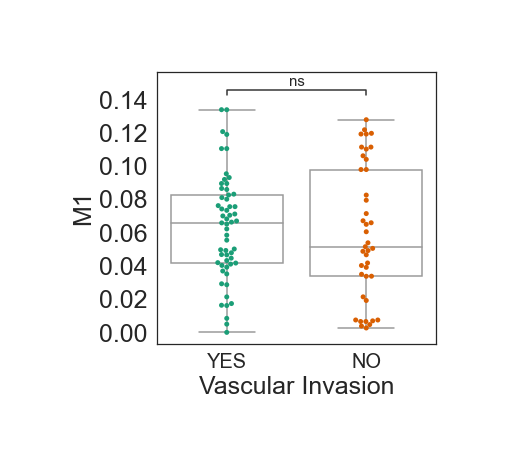
C)
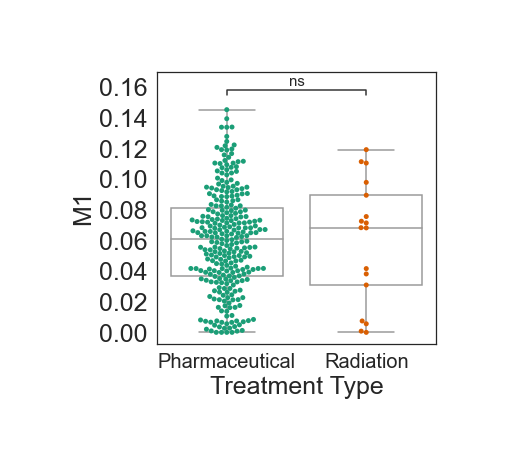


D)
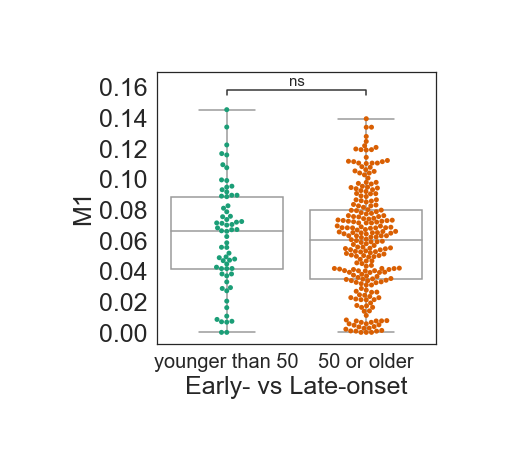
E)
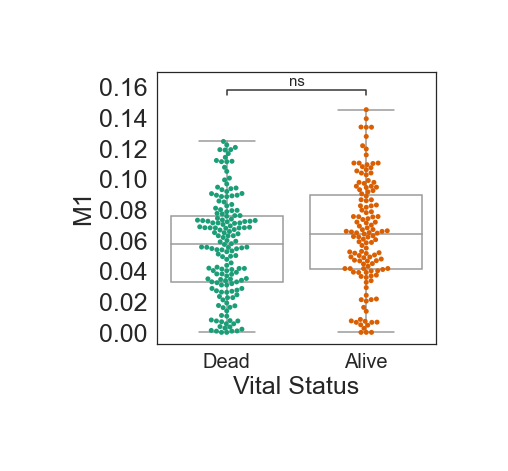
F)
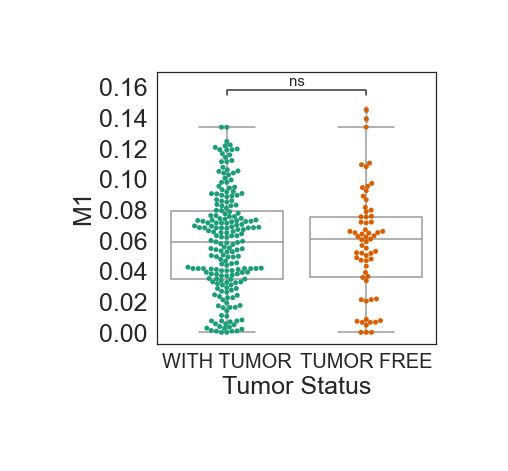


Supplemental Figure 16: Associations Between M1 Macrophages and Clinical Features. A) Lymphovascular invasion. B) Vascular Invasion. C) Treatment Type. D) Early- vs Late-onset. E) Vital status. F) Tumor status.

A)
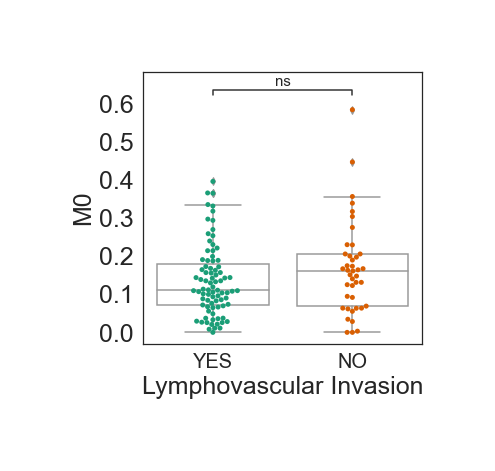
B)
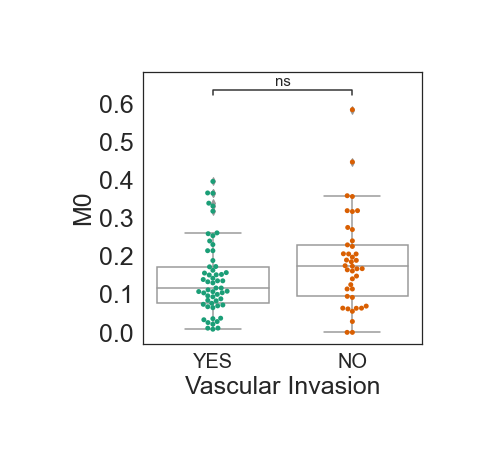
C)
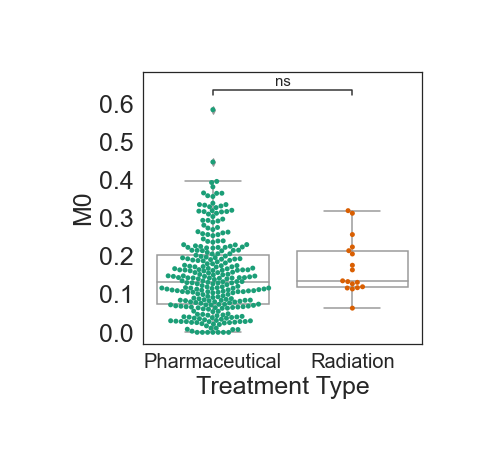


D)
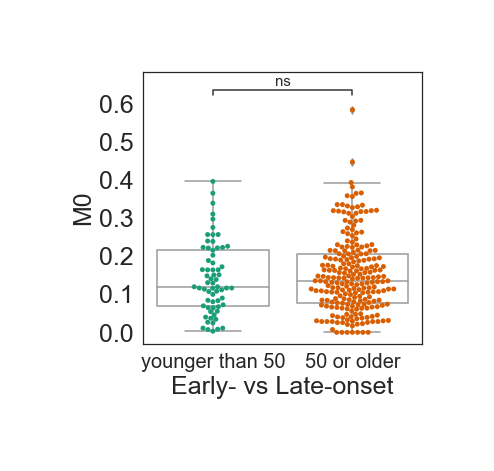
E)
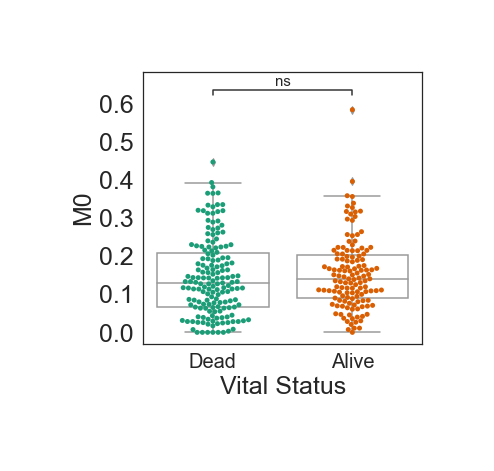
F)
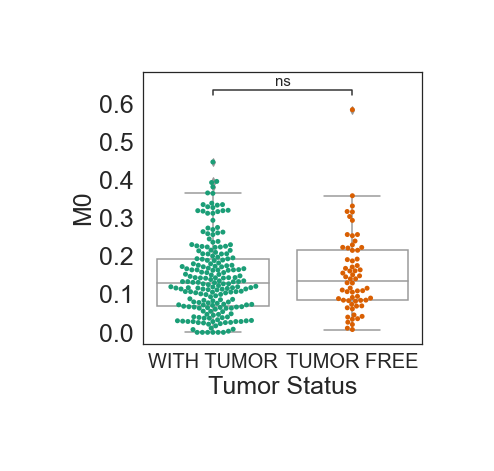


Supplemental Figure 17: Associations Between M0 Macrophages and Clinical Features. A) Lymphovascular invasion. B) Vascular Invasion. C) Treatment Type. D) Early- vs Late-onset. E) Vital status. F) Tumor status.

A)
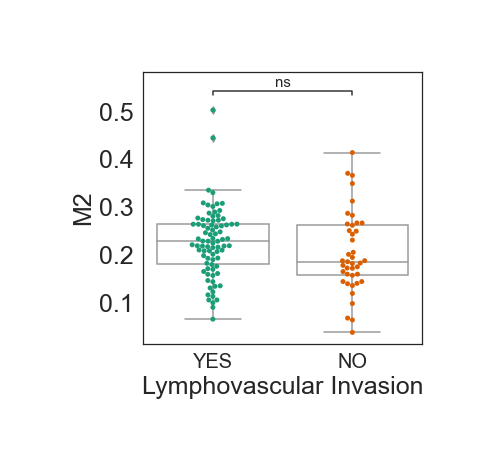
B)
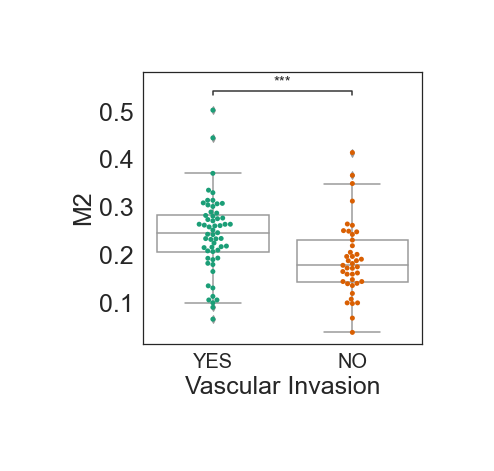


C)
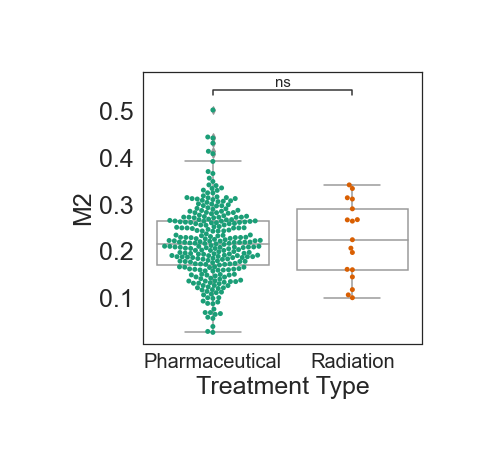
D)
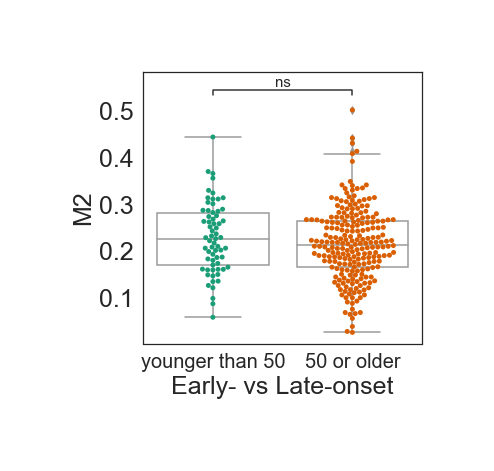


Supplemental Figure 18: Associations Between M2 Macrophages and Clinical Features. A) Lymphovascular invasion. B) Vascular Invasion. C) Treatment Type. D) Early- vs Late-onset.

A)B)C)

D)E)F)

Supplemental Figure 19: Associations Between CD4+ T Cells and Clinical Features. A) Lymphovascular invasion. B) Vascular Invasion. C) Treatment Type. D) Early- vs Late-onset. E) Vital status. F) Tumor status.

A)B)C)D)

E)F)G)H)

I)J)K)L)

M)N)O)P)Q)R)S)

Supplemental Figure 20: Kaplan–Meier Survival Analysis Based on Immune Cell Ratios using median values as cut off. A) CD4+ T Cells, B) CD8/CD4 ratio, C) CD8/Tregs ratio, D) MI/M2 ratio, E) Neutrophils, F) Eosinophils, G) T Gamma Delta, H) CD4+ naive T cells, I) Mast cells, J) Dendritic cells, K) Monocytes, L) CD8+ T cells, M) NK cells, N) Tregs, O) B cells, P) Plasma cells, Q) M1 Macrophages, R) M0 Macrophages, S) M2 Macrophages

A)B)C)D)E)F)G)H)I)J)K)L)

M)N)O)P)Q)R)

Supplemental Figure 21: Kaplan–Meier Survival Analysis Based on Immune Cell Ratios using mean values as cut off. A) CD8/CD4 ratio, B) CD8/Tregs ratio, C) MI/M2 ratio, D) Neutrophils, E) Eosinophils, F) T Gamma Delta, G) CD4+ naive T cells, H) Mast cells, I) Dendritic cells, J) Monocytes, K) CD8+ T cells, L) NK cells, M) Tregs, N) B cells, O) Plasma cells, P) M1 Macrophages, Q) M0 Macrophages, R) M2 Macrophages

A)B)C)D)E)F)G)H)I)J)K)L)

M)N)O)P)Q)R)S)

Supplemental Figure 22: Kaplan–Meier Survival Analysis Based on Immune Cell Ratios using upper quantile values as cut off. A) CD4+ T Cells, B) CD8/CD4 ratio, C) CD8/Tregs ratio, D) MI/M2 ratio, E) Neutrophils, F) Eosinophils, G) T Gamma Delta, H) CD4+ naive T cells, I) Mast cells, J) Dendritic cells, K) Monocytes, L) CD8+ T cells, M) NK cells, N) Tregs, O) B cells, P) Plasma cells, Q) M1 Macrophages, R) M0 Macrophages, S) M2 Macrophages

A)B)C)D)E)F)G)H)I)J)K)L)M)N)O)P)Q)R)S)

Supplemental Figure 23: Kaplan–Meier Survival Analysis Based on Immune Cell Ratios using lower quantile values as cut off. A) CD4+ T Cells, B) CD8/CD4 ratio, C) CD8/Tregs ratio, D) MI/M2 ratio, E) Neutrophils, F) Eosinophils, G) T Gamma Delta, H) CD4+ naive T cells, I) Mast cells, J) Dendritic cells, K) Monocytes, L) CD8+ T cells, M) NK cells, N) Tregs, O) B cells, P) Plasma cells, Q) M1 Macrophages, R) M0 Macrophages, S) M2 Macrophages

Supplemental Figure 24: Overview of immune compositions per clusters. A) shows the stacked bar chart of the immune cells’ fractions per patient. B) shows the average frequencies of immune cells in 4 clusters obtained from K-means clustering. C) PC2–PC3 projection of all clusters. D) PC1–PC2- PC3 projection of all clusters. E) The box plots of patients’ days to death in each cluster. F) The box plots of patients age in each cluster.

A)B)

C)D)

E)

Supplemental Figure 25: Clinical features per cluster. A-C Barplot of clinical features per cluster, A) Neoplasm Histologic Grade, B) Largest residual nodule, C) Primary tumor site. D) Bar plots of patients’ vital status portion vs Figo Stage. E) Boxplot of patients’ immune score vs vital status.

A)B)

C)D)

E)F)

Supplemental Figure 26: Kaplan-Meier survival analysis and stage-wise vital status. Sub-plots A and B show overall survival across 4 clusters after 5 and 4 years respectively. Sub-plots C and D shows survival curves for patients in stage III and stage IV across 4 clusters respectively. Subplots E and F show survival curves based on figo stage.
